# Review of mendelian randomization studies on age at natural menopause

**DOI:** 10.3389/fendo.2023.1234324

**Published:** 2023-09-11

**Authors:** Xiao Zhang, Zhao Huangfu, Shaowei Wang

**Affiliations:** ^1^ Department of Obstetrics and Gynecology, Beijing Hospital, National Center of Gerontology, Beijing, China; ^2^ Institute of Geriatric Medicine, Chinese Academy of Medical Science, Beijing, China; ^3^ Graduate School of Peking Union Medical College, Peking Union Medical College, Chinese Academy of Medical Sciences, Beijing, China; ^4^ Department of Urology, Changhai Hospital, Naval Medical University, Shanghai, China

**Keywords:** age at natural menopause, early menopause, late menopause, mendelian randomization, genome-wide association study, causality

## Abstract

Menopause marks the end of the reproductive phase of life. Based on epidemiological studies, abnormal age at natural menopause (ANM) is thought to contribute to a number of adverse outcomes, such as osteoporosis, cardiovascular disease, and cancer. However, the causality of these associations remains unclear. A powerful epidemiological method known as Mendelian randomization (MR) can be used to clarify the causality between ANM and other diseases or traits. The present review describes MR studies that included ANM as an exposure, outcome and mediator. The findings of MR analyses on ANM have revealed that higher body mass index, poor educational level, early age at menarche, early age at first live birth, early age at first sexual intercourse, and autoimmune thyroid disease appear to be involved in early ANM etiology. The etiology of late ANM appears to be influenced by higher free thyroxine 4 and methylene tetrahydrofolate reductase gene mutations. Furthermore, early ANM has been found to be causally associated with an increased risk of osteoporosis, fracture, type 2 diabetes mellitus, glycosylated hemoglobin, and the homeostasis model of insulin resistance level. In addition, late ANM has been found to be causally associated with an increased systolic blood pressure, higher risk of breast cancer, endometrial cancer, endometrioid ovarian carcinoma, lung cancer, longevity, airflow obstruction, and lower risk of Parkinson’s disease. ANM is also a mediator for breast cancer caused by birth weight and childhood body size. However, due to the different instrumental variables used, some results of studies are inconsistent. Future studies with more valid genetic variants are needed for traits with discrepancies between MRs or between MR and other types of epidemiological studies.

## 1 Introduction

The definition of menopause, 12 months of amenorrhea, marks the end of the reproductive phase of life (1). Menopause typically occurs between the ages of 49 and 52 years old (2). Early menopause (EM) occurs between the ages of 40 and 45. Late menopause (LM) occurs after the age of 55 (3). Primary ovarian insufficiency (POI) is considered to be present when a woman who is less than 40 years old has had amenorrhea for 4 months or more, with two serum follicle-stimulating hormone levels (obtained at least 1 month apart) in the menopausal range, which was previously referred to as premature menopause or premature ovarian failure (4). It has been reported that 11% of women experience POI or LM (5). Certain epidemiological studies have reported that women who experience POI or EM have an increased risk of morbidity and mortality in later life, including cardiovascular disease (6), osteoporosis (7), depressive symptoms (8), and type 2 diabetes (9). However, women who experience LM are more likely to have hyperlipidemia (10), hypertension (11), cerebrovascular disease (12), breast cancer (13), ovarian cancer (14), and endometrial carcinoma (15). Thus, abnormal age at natural menopause (ANM) (16), the cessation of menses for 12 months without any medical interventions, is a significant issue for a woman’s quality of life and health. To improve the care of women with this condition, it is essential to have a thorough understanding of the risk factors (causes) and the adverse outcomes (consequences) of ANM.

Although randomized controlled trials (RCTs) are the gold standard for determining a causal relationship between disease and treatment, observational studies are frequently chosen to due to RCT’s frequently high cost, time requirements, relatively small sample size, high failure rate and limited studies phenotypes (17). Although observational studies can provide information regarding the relationship between disease exposure and outcome, it is frequently challenging to prevent the influence of confounding factors and reverse causality and these studies therefore cannot prove causality (18). To limit the constraints of RCT and observational research, Mendelian randomization (MR), which is based on the results of genome-wide association studies (GWAS), has been extensively utilized to examine the causality between exposures and outcomes. The goal of MR research is to identify genomic locus variations linked to complex traits in the population, in particular, to identify the associations between single nucleotide polymorphisms (SNPs) and common diseases (19). There are two features that make MR advantageous for this type of study. Firstly, alleles are allotted at random during meiosis, and are frequently unaffected by environmental or lifestyle influences. Secondly, genetic variation can typically be detected and reported properly due to the ongoing advancements in sequencing and analysis technologies (20).

**Figure 1:**
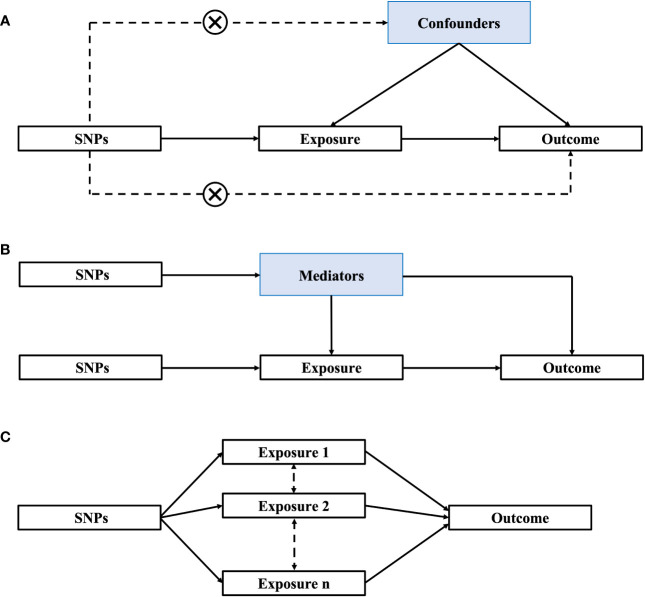
Methods of Mendelian randomization (MR). **(A)** One-sample/Two-sample MR; **(B)** Mediation MR; **(C)** Multivariable MR. SNPs, single nucleotide polymorphisms.

The use of MR is based on the following three premises: (1) genetic instrumental variables (IVs) are consistently related to the relevant risk factors; (2) genetic variants are not related to confounders of the risk factor-outcome association; and (3) genetic variants only influence the outcome through the relevant risk factor (**Figure 1A**). Current MR methods can be divided into one-sample, two-sample, mediation and multivariable MR. One-sample and two-sample MRs assess the causal relationship between exposure and outcome by using one or two independent cohorts (21). Two-sample MR results are more conservative and have a lower false-positive rate than one-sample MR since there is no bias due to weak instrumental variables (22). Mediation analysis can identify factors (mediators) that affect the relationship between exposure and outcome (21) (**Figure 1B**), while multivariable Mendelian randomization (MVMR) can simultaneously analyze the direct causal effects of each of the correlated exposures in a single analysis (23). MVMR can also simultaneously quantify the direct effects of exposure trait and potential mediator on the outcome (**Figure 1C**).

**Figure 2:**
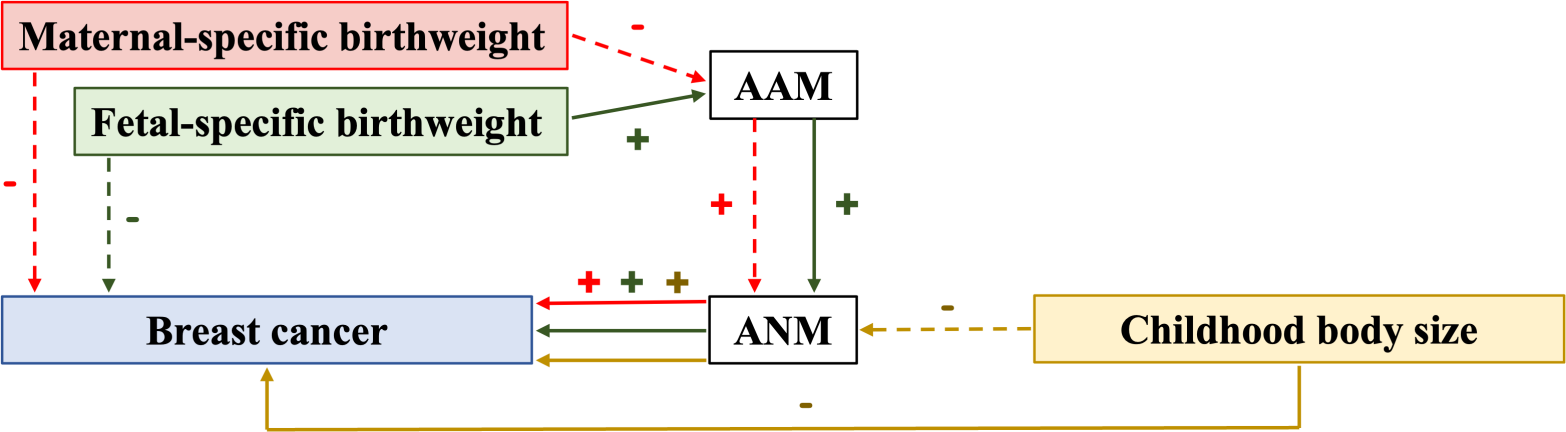
Relationship with evidence of causation identified in mediation MR studies. AAM, age at menarche; ANM, age at natural menopause; (+) indicates a positive relationship; (–) indicates a negative relationship; solid arrow indicates the presence of association; dot arrow indicates the absence of association; the color of arrows, lines, and symbols is consistent with that for maternal-specific birthweight, fetal-specific birthweight, and childhood body size.

Although previous epidemiological research has identified a wide range of causes and consequences related to EM and LM, causality remains largely undefined. Numerous recent MR studies with an emphasis on ANM have been conducted and have challenged the results of some previous epidemiological studies. Thus, it is crucial to monitor research developments and highlight the quality and effectiveness of MR. In the present review, the published MR studies involving ANM are summarized. Several studies examined ANM as the outcome (**Supplementary Table 1**), while others examined ANM as the exposure (**Supplementary Table 2**). Certain studies also demonstrated that ANM can be a mediator (**Figure 2**).

## 2 Search strategy and selection criteria

**Figure 3:**
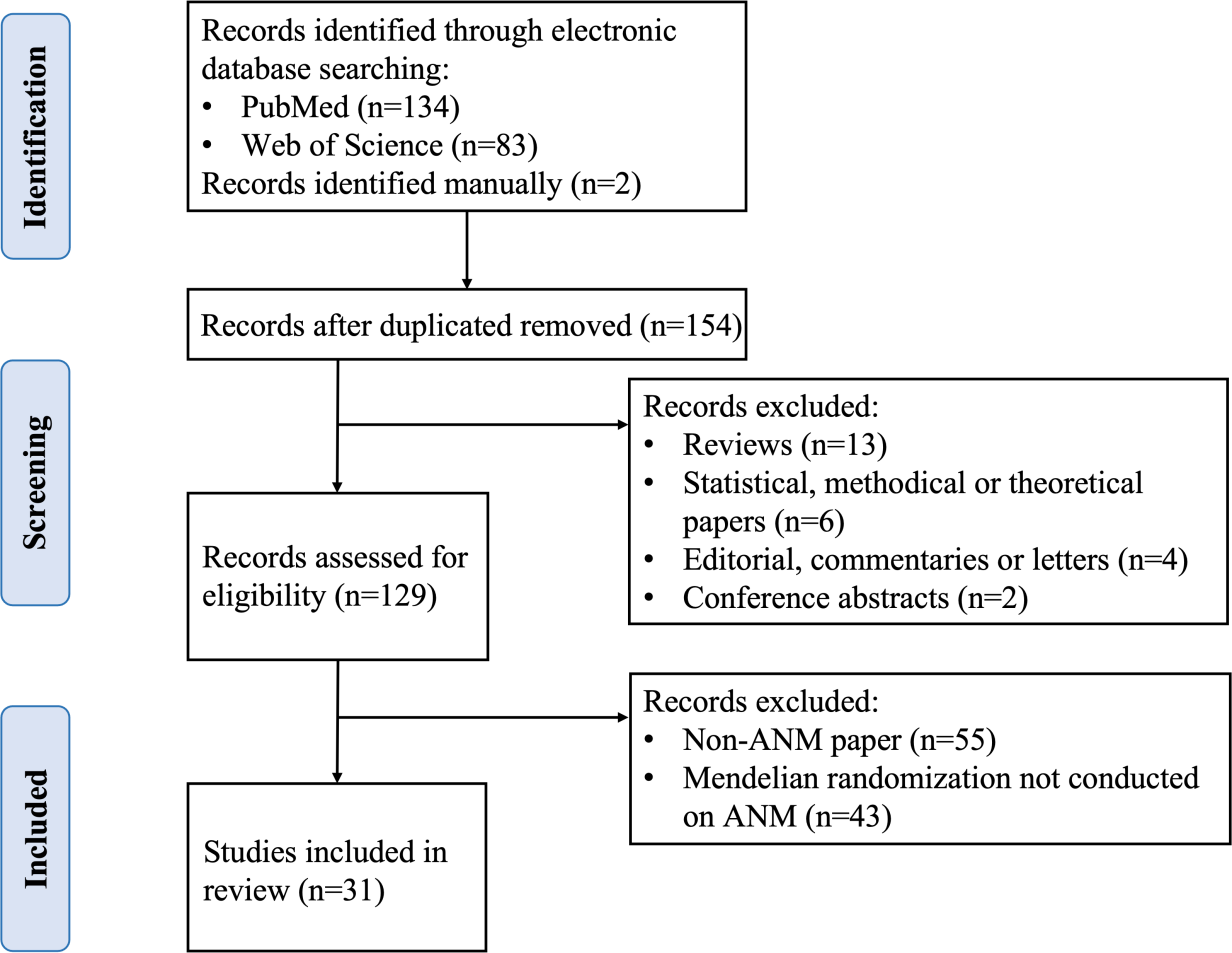
Study selection of literature search. ANM, age at natural menopause.

Original studies published before March 1, 2023 were identified by searching for relevant articles in the PubMed and Web of Science databases. The following search criteria were used, with no restriction on subheadings: “mendelian randomization” or “genetic instrumental variable” or a relayed term (e.g., “genetic instrument”) and “premenopausal” or “early menopause” or “perimenopausal” or “premature menopause” or “primary ovarian insufficiency” or “menopause” or “late menopause” or “natural menopause” or “postmenopause” or “age at menopause” or “menopausal age”. All retrieved articles were checked for relevant citations and studies not retrieved in the above electronic searches were searched manually. Studies that assess the risk factors or consequences of abnormal ANM using MR methods and IV analysis were included. Two authors (XZ and ZH) independently retrieved and reviewed studies using the search technique and selection criteria. If necessary, a third author (SW) evaluated any inconsistencies that arised. Ultimately, 31 articles were identified (**Figure 3**).

## 3 ANM as an Outcome

Since some risk factors are adjustable, such as smoking, education level, and obesity, identifying risk factors for ANM offers hope for future prevention of abnormal ANM-related diseases.

### 3.1 Smoking

Although numerous studies have found that smoking is a risk factor for POI and EM (24, 25), and although some underlying mechanisms have been identified (26), for example, increased oocyte apoptosis, increased estrogen clearance, or increased adrenalin androgen secretion, no statistically significant association between current smoking habit and EM was detected in the MR analysis by Ding et al. (β=0.26, se=1.46, p>0.05) (27). A plausible explanation for these opposing results may be that the risk of EM in smokers is dose- and duration-dependent. As one study demonstrated, only individuals who smoked for >26 years or who smoked at least 10 cigarettes a day showed a significant increased risk of EM (28). However, since the SNPs included in the MR analysis were not classified according to the duration and dose of smoking, the statistical significance of the pooled-effect estimate may have been diminished (28). Therefore, a more comprehensive MR analysis should be performed, including other smoking indicators such as previous smoking and/or number of cigarettes smoked per day (28, 29). Furthermore, mediation MR could be utilized to explore the mediators that may play a bridging role between smoking and ANM, to improve understanding of the relationship between smoking and EM.

### 3.2 Education

In epidemiological studies, there is a debate concerning the relationship between educational attainment and ANM. Certain studies have revealed that women with more years of schooling have a later menopause (2, 30), while others revealed the opposite (31, 32). However, one study found that there was no relationship between educational attainment and ANM (33). The discrepancy between these findings may be due to a number of methodological factors, including the likelihood of bias, insufficient adjustment for confounding factors, and small sample size. A two-sample MR study by Ding et al. demonstrated that a lower educational level was causally associated with EM, indicating that EM was less common in individuals with a genetic predisposition for longer education (27). This finding supported a causal relationship between lower education level and EM, based on eliminating the potential bias of confounding factors, such as a high-earning job, wealth, high social status, and awareness of healthy habits, present in traditional observational studies. Thus, new evidence was provided for further research on the mechanism of education level or brain cognition on ANM. The biological plausibility of an association between educational level and menopause might be hidden in the complex mechanisms of how the brain functions, which warrants further investigation (34).

### 3.3 Obesity

Based on the results of both observational and MR studies, the impact of obesity on ANM remains inconclusive. A large prospective study found that women who were underweight (body mass index, BMI<18.5 kg/m^2^) had an increased risk of EM, while women who were overweight (BMI 25-29.9 kg/m^2^) or obese (BMI≥30 kg/m^2^) tended to experience LM (35). This effect was not only observed for adult weight, but also for childhood weight (36). This suggested that obesity has a protective effect on EM, possibly through the higher production of estrone in adipose tissue (30, 37). However, other studies support the opposite conclusion (38). Moreover, the association between body weight and ANM is not significant when adjusting for reproductive factors or smoking (38, 39). Nonetheless, self-reported BMI, moderate heterogeneity between studies, and potential confounders, such as smoking, may have an impact on these findings.

By contrast, the genetic risk score (GRS) for adult BMI increasing variants with BMI profiles from early to late adulthood showed that this association was highest in women with EM (40). Higher BMI was linked to a higher risk of EM in an MR study conducted by Ding et al, but lipid level, including total cholesterol and low-density lipoprotein, had no significant effect on ANM (41). This suggested that the adipose tissue of obese women may cause EM through a different mechanism (27). However, Ardissino et al. did not find any evidence of an association between BMI and ANM (41). In addition, bidirectional MR analysis did not find any evidence of abnormal ANM causing abnormal BMI or percentage body fat, clarifying the direction of causation (41–45). These contradictory findings are likely the result of different SNP selections and different sample sizes. Given the possible complex link between adipose tissue and hormones, the effect of obesity or emaciation on ANM needs further study.

### 3.4 Female reproductive factors

The reproductive life course of a woman includes the age at menarche (AAM), ANM, the age at which she starts and stops having children, and the number of children she has, as well as the age she first has sexual intercourse and the number of sexual partners she has during her lifetime. These factors are often intertwined and influence each other. Some studies have reported that early AAM (<11 years of age) is significantly associated with EM, while later AAM (>13 years of age) leads to an increased risk of LM (30, 46). In addition, early menarche without childbearing further increases the risk of EM (~2-fold) and POI (5-fold) (47). Furthermore, age at first birth (AFB), a late first birth, more induced abortions, and longer breastfeeding are inversely associated with POI and/or EM (30, 48).

In 2015, Day et al. identified a genetic correlation between AAM and ANM (42). SNPs related to AAM and ANM are located in or near genes axis such as *CHD7*, *FGFR1*, *SOX10*, *KISS1* and *TAC3*, indicating that both reproductive milestones are at least partially governed by shared biological mechanisms (42, 49). In addition, a positive genetic correlation between AAM, AFB or age at first sexual intercourse (AFS) and ANM has also been confirmed by MR studies, independent of childhood body size (27, 50, 51). However, other female reproductive factors, including lifetime number of sexual partners, ever being parous, number of live births, and age at last birth are not associated with ANM (50). This might be the result of numerous interactions between reproductive factors spaced widely apart over time, which might be mediated by other reproductive events in between. However, reverse MR analysis demonstrated that ANM did not significantly affect other reproductive factors, indicating the direction of causality (50).

The reason why earlier AAM results in earlier subsequent reproductive events, including ANM, may be explained by the life history theory. According to this theory, life history strategies can be divided into “fast” or “slow” (52, 53). A “fast” life history strategy puts more effort into reproduction, resulting in earlier puberty and sexual activity, an early AFB, and an increased number of births (52, 53). A “fast” life history may result in a younger AAM due to the allocation of resources towards earlier reproductive endeavors, and a higher number of children, leading to the completion of reproduction at a younger age. Another explanation is that the factors, like maternal smoking during pregnancy or low protein intake reported during childhood, that cause early AAM could influence the oocyte pool (54). These factors could also affect follicle quantity and quality, and the rate of follicle decline after birth, which results in a decrease in the follicle pool, ultimately leading to the early onset of menopausal events (36).

### 3.5 Thyroid function

Normal thyroid function plays a significant role in normal sexual function (55). Previous studies have found that Hashimoto’s thyroiditis (or thyroid autoimmune disease) was closely related to ANM (56). To clarify whether thyroid function, including the function of thyroid stimulating hormone (TSH), free thyroxine 4 (fT4), hypothyroidism and hyperthyroidism is causally associated with sex hormones and sexual function, including ANM, Kjaergaard et al. conducted an MR analysis (57). It was determined that TSH-related SNPs, which are associated with autoimmune thyroid disease (AITD), were associated with EM. It was also found that the GRS for fT4 was associated with LM. However, TSH, fT4 restricted to the *DIO1* and *DIO2* genes, subclinical hypothyroidism, subclinical hyperthyroidism, and overt hypothyroidism had no significant correlation with ANM. This result was consistent with previous observational research findings (56, 58).

It is thought that 4-30% of occurrences of EM are caused by autoimmune-related diseases (59), and thyroid-related disorders rank first among these diseases (60). Therefore, the link between thyroid diseases and abnormal ANM is likely due to an abnormal immune system. Studies concerning patients who were either positive for thyroid peroxidase antibody or anti-thyroglobulin antibodies determined a genetic correlation between serum anti-müllerian hormone (AMH) and TSH levels. In thyroid-related autoimmune diseases, autoantibodies may attack ovarian tissue, exacerbating the loss of ovarian reserve and leading to EM or even POI (61). Therefore, women with EM or POI should be considered for thyroid function and thyroid antibody testing to be alert for the presence of AITD. Women with autoimmune diseases, including AITD, should also be actively treated to prevent the occurrence of EM and its subsequent complications. Although the causal relationship between thyroid function and ANM in this MR study conducted by Kjaergaard et al. was not very robust, genetically predicted thyroid function was highly correlated with sex hormone concentrations, including sex hormone-binding globulin (SHBG) and testosterone (57). Therefore, thyroid dysfunction may also regulate ANM by altering the levels of other sex hormones. For example, thyroid hormones can increase SHBG concentration or interfere with the connection between the hypothalamic-pituitary-thyroid and the hypothalamic-pituitary-gonadal axes through prolactin (55, 62).

### 3.6 Serum homocysteine concentration and methylene tetrahydrofolate reductase gene mutation

Homocysteine (Hcy) is a chemical in the blood, which is formed when the amino acid methionine is naturally broken down to be excreted in the urine (63). Certain observational studies have identified a link between elevated plasma Hcy concentration and reduced female fertility (phases of menstrual cycle and menopause) (64, 65), while other studies have found no such connection (66, 67). To investigate whether genetically elevated plasma Hcy is related to fertility, including ANM, Kjaergaard et al. conducted a two-sample MR study based on a large meta-GWAS dataset (68). It was found that only the functional variant, methylene tetrahydrofolate reductase (*MTHFR*) rs1801133, was associated with 7.45 months ANM delay, of the 18 genetic variants associated with Hcy or related diseases. This causal association was no longer significant after the addition of other related SNPs, which implies that there is no association between genetically elevated Hcy and ANM. In the process of biological metabolism, the *MTHFR* gene is significantly related to Hcy concentration, but the interference of *MTHFR* gene variants on ANM may not be achieved by affecting the Hcy pathway (67). Further studies using independent samples on a larger scale and with different ethnicities are required to comprehend the links between the *MTHFR* gene and the onset of menopause.

### 3.7 Psychiatric disorders

There may be a complicated link between reproductive behavior and psychiatric disorders. Patients with psychiatric disorders and their relatives may be more likely to engage in risk-taking and impulsive behaviors, which may lead to early pregnancy and childbirth, or these patients may have poor social skills, which may delay important reproductive transitions such as marriage, pregnancy, and childbirth, resulting in the occurrence of abnormal ANM (69, 70). However, the MR study conducted by Ni et al. did not find a potential causal relationship between ANM and some psychiatric disorders (such as attention-deficit/hyperactivity disorder, bipolar disorder, and schizophrenia) associated with early reproductive events such as AFB and AFS (51). This may be due to the underlying causal mutation of psychiatric disorders being more likely to affect reproductive success than reproductive ending events.

## 4 ANM as an Exposure

To effectively counsel affected women in the avoidance of these diseases, a thorough awareness of the genuine effects of EM and LM is necessary.

### 4.1 Cardiovascular disease risk

The primary cause of morbidity and mortality in women is cardiovascular disease (CVD) (71). Observational studies have recently discovered that EM is associated with later life CVD in women, especially in women who have undergone bilateral oophorectomy but have not received hormone replacement therapy (HRT) (72–74). The use of a weighted GRS (wGRS) also confirmed that EM may significantly increase the risk of coronary heart disease-related death (44). It has been postulated that this effect is due to the cardioprotective effects of estrogen (75). However, MR studies have not found evidence of a potential causal relationship between EM and a number of cardiovascular diseases, including atrial fibrillation, coronary artery disease, heart failure, ischemic stroke, stroke and its subtypes (small vessel stroke, large-artery atherosclerotic stroke, and cardioembolic stroke), as well as some cardiovascular disease risk factors, such as cholesterol levels, fasting glucose, glycosylated hemoglobin A1c, C reactive protein and apolipoprotein levels (41, 76–78). There are several plausible explanations for these differences in results between studies using different methods. The discrepancy between the wGRS and MR findings may be due to collider bias, a bias associated with the genetic variant and the outcome through confounders, or the small sample size of the wGRS study (79). Differences between observational studies and MR analyses may also be related to residual confounders (age or long-term HRT) or survivor bias in GWAS (the presence of women who died from cardiovascular events before menopause) (80). Therefore, ANM may not have a causal relationship with CVDs, or variants associated with both ANM and CVDs might not have been found yet. In addition, the GWAS study on ANM did not include women with POI, but most observational studies and meta-analyses did include these women, and the results indicated that POI is associated with both fatal and non-fatal coronary heart disease and CVDs (42, 43, 81, 82). Therefore, it may be POI rather than EM that increases the risk of CVDs. Furthermore, reverse causation could be a potential problem in observational studies. Although the majority of researchers believe that EM increases the risk of CVDs, the association can also be observed due to a poor cardiovascular risk profile or accelerated vascular aging causing EM. A previous study demonstrated that women who have CVDs before the age of 35 years old experience accelerated menopause (80). However, no reverse association of CVDs or dyslipidemia with an increased risk of EM was found in observational or MR studies (76, 83). Given the inconsistencies in the current findings, a stratified analysis of ANM data from a larger cohort, including patients with POI, is needed to confirm whether a causal association between POI/EM and CVDs exists, and the operability of HRT to reduce the risk of postmenopausal CVDs.

### 4.2 Hypertension

Hypertension is the primary modifiable risk factor for CVDs and mortality (84). Currently, studies have demonstrated an inconsistent correlation between ANM and blood pressure traits. Certain studies have shown that EM may increase hypertension and subsequent risk of CVDs in several populations, including Caucasian, Korean, and Chinese people (6, 85–88), while another study has demonstrated a positive association between LM and hypertension (89). To clarify inconsistent results caused by the influence of confounding factors and reverse causality in observational studies, Roa-Diaz et al. (90) conducted an MR analysis based on data from a CoLaus study (91) and a Rotterdam study (92). It was found that a 1-year increase in ANM was related to a 0.45 mmHg increase in systolic blood pressure (SBP), based on the Rotterdam Study-III-1. However, after the exclusion of women who reported using HRT, EM was associated with lower SBP and diastolic blood pressure, as well as a lower risk of hypertension. In addition, Roa-Diaz et al. (90) did not find any evidence of a causal association between ANM and blood traits in a one-sample MR regardless of whether antihypertensive medication was adjusted, but a potential positive causal association between ANM and SBP was observed in a two-sample MR analysis. Considering that two-sample MR findings are more rigorous, Roa-Diaz et al. believed that there is no notable correlation between ANM and hypertension, but the relationship between ANM and SBP is worth further investigation. More notably, as SNPs restricted to DNA damage response (DDR) genes showed statistical significance with SBP, Roa-Diaz et al. proposed that LM may induce the development of high SBP through the DDR pathway. The induction of this pathway may be long-term exposure to estrogen or regulation by some of the stress hormones that are affected by menopause (93, 94). However, this theory has not yet been validated and, as DDR variants have not been linked to blood pressure traits, the role of DNA repair in blood pressure is not well understood. However, the results of the MR study conducted by Roa-Diaz et al. (90) still provide a new direction for future research, that is to evaluate the links between DDR pathways, sex hormone and the ageing process, as well as their role in the onset of menopause and progression of CVDs in women.

### 4.3 Type 2 diabetes mellitus

Type 2 diabetes mellitus (T2DM) is one of the primary causes of death and CVDs in the world (95). At present, the correlation between ANM and T2DM is controversial (96–99). The inconsistencies in the results are possibly caused by variations in variable adjustment, ethnicity diversity in the sample population, or genetic traits. Additionally, due to the limitations of existing knowledge, there are potential or unidentified confounding factors, such as smoking, physical activity, and some social factors, that cannot be adjusted for (100–102). To prevent the interference of potential confounding factors and the impact of reverse causality, several MR studies were conducted to elucidate the possible causal association between ANM and T2DM. In these MR analyses, none of the studies found a causal association between ANM and T2DM, fasting glucose, fasting insulin, or the homeostasis model of B-cell function (76, 103, 104), except for a recent GWAS of ANM that noted a link between the genetic risk for EM and T2DM (43). Notably, the causal association between EM and the levels of glycosylated hemoglobin A1c (HbA1c) is disputed (76, 103), but a causality between EM and a higher homeostasis model of insulin resistance (HOMA-IR) level has been confirmed (104). These findings could also reflect the possibility of EM leading to an abnormal elevation of IR, thus increasing the risk of T2DM. This relationship is typically attributed to alterations in sex hormone levels, primarily due to decreased estrogen levels after menopause that diminish the protective effect to islet cells (105). However, the exact pathogenetic mechanisms underlying the association between EM and T2DM risk or increased IR cannot be fully elucidated. A possible mechanism is a reduction in the role of estrogen receptor α, which binds to β-cells to regulate insulin biosynthesis and secretion and β-cells survival, due to the shortened duration of estrogen exposure (106). In addition, estrogen deficiency can cause glucose intolerance and body fat redistribution, leading to increased central adiposity and increased IR (107, 108). Furthermore, menopause-related changes in serum concentrations of other steroid hormones, such as testosterone, as well as SHBG, may also play a role in the development of T2DM (109, 110). Despite all of this, whether the insignificant effect of ANM on glucose metabolism is masked by a compensatory increase in insulin or whether it is truly irrelevant remains unclear, attention should still be paid to the influence of EM on islet cells and blood glucose.

### 4.4 Breast cancer

Breast cancer (BC) is the most prevalent cancer among women globally and the leading cause of cancer-related death in women (111). Recently, the role of ANM in the incidence of BC has received increasing attention, but the results are often contradictory (112–114). According to the results of our literature search, a total of 7 included studies have verified the causal relationship between ANM and BC by MR analysis (43, 78, 103, 115–118). However, the results were not completely consistent as each study used different GWAS data or selected a various number of SNPs. Chen et al. reported that older ANM was significantly associated with an elevated risk of all intrinsic subtypes of BC, including estrogen receptor (ER)+, ER-, luminal A-like [ER+ and/or progesterone receptor (PR)+, human epidermal growth factor receptor 2 (HER2)-, grade 1 and 2], luminal B-like (ER+ and/or PR+, HER2+), HER2-enriched-like (ER- and PR-, HER2+) BC, except triple-negative BC (ER-, PR-, HER2-) (115). Jia et al. (117), Si et al. (118), Magnus et al. (103), and Ruth et al. (43) also found a positive association between ANM and BC. However, Escala-Garcia et al. (116) and Lankester et al. (78) did not find any casual association between ANM and BC including ER+ and ER- BC. After ruling out small SNPs available and overlapping populations, Escala-Garcia et al. speculated no causality was observed due to selection bias. This type of collider bias can result in an under- or overidentification of genetic risk factors for BC survival due to a correlation between the genetic risk factor in question and BC incidence (119).

Although the results of the MR studies were inconsistent, the hypothesis that LM causes a higher risk of BC (except for triple-negative BC) is still accepted by most scholars. The basic hypothesis behind this consideration is that long-term exposure to estrogen may affect the behavior of cells, which then develop into cancer cells over time. Notably, there is evidence that ovariectomy (early surgical menopause) can decrease the risk of breast cancer (120). Therefore, women with LM should be advised to perform BC screening tests earlier given the association of a higher risk of BC. In addition, both observational and MR studies demonstrated that ANM and SHBG had no significant association with triple-negative BC (113, 115, 121). Therefore triple-negative BC may have a different etiological profile than other BC subtypes, and the prevention and intervention of triple-negative BC should not be treated in accordance with other subtypes of BC.

### 4.5 Endometrial cancer

Endometrial cancer (EC) is the most prevalent invasive gynecologic cancer in women living in developed countries, and is also the most common type of uterine cancer (122). Some studies have suggested a significant association between LM and an increased risk of EC (123, 124), but the effect size in different studies was variable, whereas no significant association was found in the other studies (125, 126). In a study by Lankester et al., both observational studies based on the Women’s Health Initiative (WHI) and the UK Biobank (UKB), and a one-sample MR analysis based on the WHI found that LM was associated with a higher risk of EC, but the evidence was not very robust (78). The authors suggested that the inconsistent results based on different databases may be due to the fact that SNPs in the IV were not as well imputed in some datasets, which could reduce the accuracy for these two-sample MR replication datasets and subsequently influence the statistical power. Therefore, Lankester et al. still supported the hypothesis that LM may increase the risk of EC (78). In addition, the impact of ANM on the 2 types of EC (type 1 - estrogen-dependent, type 2 - estrogen-independent) cannot be determined respectively as the EC subtypes were not analyzed by stratified analysis. Considering that ANM is mainly related to changes in sex hormones, ANM may only lead to an increased risk of type 1 EC, and therefore may have no significant association with type 2 EC. The use of an overall EC containing both types may be the cause of inconsistent results. Therefore, future studies should analyze the different types of EC separately, to better determine the effects of ANM on different types of EC and to improve the prevention of specific types of EC. In addition, two-sample MR or MVMR with higher quality should be considered to explore the association between ANM and EC.

### 4.6 Ovarian cancer

Ovarian cancer (OC) is the second most prevalent cancer of female reproductive system and the primary cause of reproductive cancer-related death (127). Epithelial OC (90%) is the most prevalent form of OC, which can be divided into serous, endometroid, clear cell, mucinous and unspecified OC (128). Several studies have shown a direct relationship between LM and the risk of OC (129, 130). Women with a ANM >52 years of age have ~2-fold increased risk of developing OC compared with women who experienced menopause at ≤44 years old (131). The association between the GRS for ANM and the risk of OC in UKB was also highlighted in the recent GWAS of ANM (43). In studies by Yarmolinsky et al. (132) and Si et al. (118), a positive association between endometrioid OC and LM was found, but no causal relationship between ANM and invasive epithelial OC or other subtypes, including high grade serous OC, low grade serous OC, clear cell OC, and low malignant potential OC, was found. These findings were consistent with another study (133). In the study by Lankester et al., a 5-year increase in ANM was associated with a greater risk of OC in observational analyses based on the WHI and UKB combined data, but no causal association between ANM and OC was discovered in MR analyses (78). These results may be due to the authors using overall OC instead of conducting a separate analysis for each OC subtype. Thus, it can be seen that endometrioid OC may be more closely related to sex hormones or other endocrine factors, and is therefore more easily be affected by some reproductive factors. It is necessary to screen for endometrioid OC in women with LM, and it is also important to further explore the underlying mechanism between the two.

### 4.7 Colorectal cancer

Colorectal cancer (CRC) is the third most frequent cancer in the world (134). Previous studies have found that women with an ANM of >49 years old had a decreased risk of CRC mortality compared with women who experienced menopause at ≤49 years old, although this protective effect was not statistically significant (135). However, in a large prospective cohort study with >214,000 postmenopausal women without a history of menopausal hormone therapy (MHT) use, women with LM had a 1.5 times increased risk of CRC compared with women with POI (136). Considering that these inconsistent results may be influenced by confounding factors, such as weight or HRT, Neumeyer et al. conducted an MR study and did not find any evidence to support the causal association between ANM or estrogen exposure and CRC (137). Additional adjustment of the analysis by BMI, education, MHT usage, smoking, family history of CRC, and regular aspirin use, did not significantly alter the results. Certain studies speculated that the effect of estrogen on CRC may vary depending on the ER-β expression status in colorectal tissues, rather than simply on the duration of estrogen exposure (138, 139). Therefore, larger studies using data on ER-β expression in colorectal tissues are necessary to assess the true impact of ANM or estrogen on CRC.

### 4.8 Lung cancer

Lung cancer (LC) ranks the second primary cause of cancer-related death among women after BC, accounting for 13.8% of all cancer-related deaths in women in 2018 (140). Previous studies found that EM can lead to an increased risk of LC in women who smoke, and it mainly occurs in small cell histology, while in women who do not smoke, LM was associated with an increased risk of LC (141, 142). However, other studies indicated that no association was observed between ANM and LC risk (143, 144). The one-sample MR study by Lankester et al. demonstrated that LM was causally related to a higher risk of LC [odds ratio (OR)=1.35, 95%CI=1.06-1.71], but this result was contrary to the results of their observational analyses (78). This notable inconsistency suggested the observational findings may have been influenced by many confounding factors, including smoking, diet, exercise, and histological subtype (78, 141, 145), or the power of IVs in the one-sample MR may be weak.

Similar to other types of cancer, such as BC and EC, longer estrogen exposure with a later ANM may promote the transformation of lung cells or the development and progression of existing subclinical primary lung tumors. This potential mechanism has been demonstrated in both clinical and animal studies (146, 147). However, a study has also suggested that the increased risk of LC caused by EM may be due to an increased production of reactive oxygen species owing to elevated iron levels after menopause (148). Since the results of the observational and one-sample MR studies were inconsistent and had their respective shortcomings, it is essential to conduct a two-sample MR, an MVMR or a large RCT with higher quality data to confirm a direct causal relationship between ANM and LC. Additionally, considering that different histological types of LC are not subject to the same effect of estrogen (149), future research is also necessary to investigate the underlying molecular mechanisms between exogenous and endogenous sex-hormones in the formation of different histological types of LC.

### 4.9 Lung function

Even in non-smokers and individuals without signs of lung illness, reduced lung function is a significant predictor of mortality in adult females (150). Most observational studies found that post-menopausal women had a decreased forced expiratory volume in one second (FEV1) and forced vital capacity (FVC) compared with pre-menopausal women, but that there was no difference in FEV1/FVC or airflow obstruction (151–154). A large study in the UKB, however, revealed that women with EM had a decreased FEV1 and FVC as well as a higher risk of spirometric restriction (152). To explore the effect of ANM on lung function, Van et al. conducted an MR study (155). Whether ANM was classed as a categories variable or a continuous variable, it was determined that LM was associated with the lower FEV1 and FEV1/FVC, and a higher risk of airflow obstruction. However, the protective effect of EM was diminished in HRT-using and overweight women. In addition, no effect of ANM was found for FVC or spirometric restriction (155). However, the MR study conducted by Magnus et al. did not replicate this conclusion due to differences in selected GWAS data (103). Despite these inconsistencies, on the whole, the later a woman’s menopause, the lower her lung function, and the more likely she is to have airflow obstruction. At present, the biological mechanisms that underlie the link between decreased lung function and menstruation cessation are not fully known, but it may be influenced by changes in the level of circulating sex hormones and elevated insulin resistance (156). Therefore, clinicians should be alerted to the risk of poor lung function in women with LM.

### 4.10 Osteoporosis and fracture

Osteoporosis is a major clinical problem for both older men and women. Almost all bones are at risk of fracture due to osteoporosis, and osteoporotic fractures are associated with higher medical costs, impaired quality of life, physical disability, and increased mortality (157). A relationship between POI/EM and lower bone mineral density (BMD) or osteoporotic fractures has been well established from traditional observational studies (158, 159). Studies even showed that women with POI are more likely to have low BMD if they had more than 1 year delay in diagnosis, age of onset of menstrual irregularity before age 20 years, and non-compliance for HRT (160). MR analyses conducted by both Lankester et al. (78) and Magnus et al. (103) also confirmed a causally harmful effect of EM on osteoporosis and fracture. A recent GWAS of ANM also revealed this connection (43). Although the MR study by Trajanoska et al. did not find a statistically significant causal relationship between EM and fracture risk, there may be bias due to the fact that the outcome data used was not all from the European population, and did not stratify outcome data by sex (161). As estrogen deficiency adversely affects the basic multicellular units responsible for bone remodeling, it is not hard to understand that POI/EM can lead to a higher risk of fracture and osteoporosis (162). Therefore, postmenopausal women, especially women with POI/EM or abnormal menstrual cycle at a younger age, should be screened for BMD to prevent osteoporotic fractures and, if necessary, different MHTs should be selected for better prevention based on age and fracture risk score (157, 163).

### 4.11 Osteoarthritis

Osteoarthritis (OA) is the most prevalent type of joint disease worldwide (164) and has been ranked as the primary cause of disability (165, 166). Certain studies have shown that OA is more likely to occur in women who receive MHT than in women who do not, suggesting that prolonged estrogen exposure may promote OA development, possibly due to a higher BMD as a result of longer estrogen exposure (167, 168). However, no causal association between ANM and OA or two OA subtypes (hip OA and knee OA) has been found by MR analysis (169). This may be because hand OA is the OA most affected by ANM (170), but this MR study only included knee and hip OA (169). Therefore, future studies should further explore the relationship between ANM and hand OA.

### 4.12 Rheumatoid arthritis

Rheumatoid arthritis (RA) is a chronic autoimmune inflammatory disease. Women frequently experience stiffness and symmetrical joint swelling, which, if ignored or improperly managed, can have disabling outcomes and limit life expectancy (171). Some previous studies have suggested a close relationship between POI/EM and RA. EM is related to a 2-fold increased risk of RA (172, 173), and patients with juvenile idiopathic arthritis are more likely to develop idiopathic POI (174). However, Zhu et al. did not observe a connection between ANM and RA before or after excluding SNPs linked to palindromic and confounding factors or after adjusting for BMI and year of education (175). They believed that the inconsistent results of observational and MR studies may be due to confounding factors or the use of instrumental variants that are not fully representative of reproductive factors, or indeed that ANM does not have a significant impact on RA, or the interference with reverse causality. However, this MR analysis (175) only analyzed the causal association from ANM to RA, but not the association from RA to ANM, and it was conducted utilizing overall RA (a majority of which are seropositive RA, >85%) without identifying disease subsets distinguished by the presence/absence of anti-citrullinated peptide antibodies (ACPA) or rheumatic factors. Moreover, different RA subgroups may be regulated by sex hormones in different ways (176, 177). For example, certain studies suggested that EM was associated with seropositivity in women with early RA (176), and that MHT use could decrease the risk of ACPA+ RA in postmenopausal women >50 years of age, but not of ACPA- RA (177). However, the biological mechanisms underlying hormonal factors and the development of RA are still not fully understood, which may involve a complex regulation of inflammatory pathways and immune responses (178–180). Future research in this area should be designed with a larger sample size and greater power.

### 4.13 Alzheimer’s disease

Alzheimer’s disease (AD), a neurodegenerative disease, is the most common cause of dementia in the elderly population (181). Epidemiological studies have suggested that decreased estrogen levels during menopause may have a significant impact on the etiology of AD and raise the risk of cognitive impairment in women (182, 183). However, inconsistent estimates ranged from a slight increase in dementia or AD risk with EM (182–185) to an inverse relationship (186, 187) or a complete loss of statistical evidence (184, 188). To ascertain whether endogenous estrogen exposure does have a causal effect on AD susceptibility, Li et al. (45) and Lankester et al. (78) performed MR analyses. Both of the studies found no association between ANM and AD or cognitive performance (45, 78). However, the observational analysis by Lankester et al. using the WHI and UKB demonstrated that a 5-year increase in ANM was associated with a lower rate of AD (78). Despite this, the interpretation of the MR results is thought to be more reliable as all the women included from the GWAS datasets linked to ANM experienced natural menopause, excluding those who had undergone surgery, radiation, or HRT before menopause. Additionally, to reduce type I error, the ANM-related SNPs belonging to the apolipoprotein E locus were also eliminated (45). Combined with the reverse-direction MR results, ANM is neither a cause nor a consequence of AD, although the beneficial effects of estrogens on the central nervous system are biologically plausible (189–191).

### 4.14 Parkinson’s disease

Parkinson’s disease (PD) is the second most prevalent form of neurodegenerative disease worldwide, after AD (192). Similar to AD, a decrease in the estrogen level is also thought to play a significant role in the development of PD (193, 194). EM, POI and a short fertile life length (<36 years) are related to a higher risk of PD, especially in women with early artificial menopause (surgical or iatrogenic) (195–197). Ultimately, the earlier the age of menopause, the higher the risk of PD (197, 198). However, other studies have shown the reverse effects (195, 199) or no association (200, 201). To avoid incorrect results caused by biases, including residual confounding and measurement error, Kusters et al. conducted an MR analysis, and reported a negative causal association between ANM and PD risk (202). In this study it was determined that each year of delay in ANM was associated with a 7% decrease in PD risk. This may be related to the neuroprotective effects of estrogen (203). For example, estrogen therapy slows down the dopaminergic neurodegeneration of the substantia nigra and restores dopaminergic transmission (204). Therefore, HRT after the abrupt decline of estrogens caused by EM or artificial menopause may be beneficial. Future research should investigate the complex changes in this period in depth.

### 4.15 Aneurysmal subarachnoid hemorrhage

Aneurysmal subarachnoid hemorrhage (aSAH) remains a devastating disease with high mortality and morbidity (205). Studies have found that a longer exposure to both endogenous and exogenous estrogens is associated with a lower risk of aSAH (206, 207). However, the MR analysis conducted by Molenberg et al. did not observe an association between genetically determined ANM or estradiol levels and the risk of aSAH but did observe an 18% increased aSAH risk among women per 1-standard deviation increase in genetically determined SHBG levels [OR=1.18, 95%confidence interval (CI)=1.05-1.34], and a 27% decrease among women per 1-standard deviation increase in bioavailable testosterone (208). Therefore, some sex hormones with large fluctuations during menopause transition, such as SHBG and bioavailable testosterone, but not ANM, may be the true risk factors for aSAH. Certain previous studies believed that a high level of SHBG had a protective effect on blood vessels (209, 210), but in the MR analysis of Molenberg et al. (208), almost all SNPs included in the univariate analyses on SHBG had secondary (opposite) impacts on bioavailable testosterone levels. Thus, testosterone may act as a mediator between SHBG and aSAH risk. However, more research is required to fully understand the mechanisms underlying the impact of various sex hormones on the development of aSAH.

### 4.16 Polycystic ovary syndrome

Polycystic ovary syndrome (PCOS), one of the most common reproductive endocrine diseases, affects the living standard, fertility, and long-term health of patients (211). Both case-controlled and MR studies demonstrated a positive association between LM and PCOS risk (212–215). A correlation between ANM-related SNPs with higher luteinizing hormone levels may partially explain this impact (213). In addition, the newly discovered PCOS locus near RAD50 is involved in the repair of DNA double-stranded breaks, and this mechanism is consistent with the function of the ANM candidate loci, therefore supporting the important role of ANM GWAS in the DNA repair pathway and its correlation with PCOS (214, 216). However, due to PCOS occurring more frequently in women of childbearing age, there are still few studies on the relationship between PCOS and menopause. Therefore, further research is still needed to determine the specific interaction mechanisms among ANM and PCOS.

### 4.17 Biological aging rate

A relationship between LM and the biological aging rate has been proposed. Delayed reproductive aging is more common in individuals from families with a history of longevity (217). In an MR analysis, one SNP (rs11668344) significantly associated with ANM was highly correlated with epigenetic age acceleration, but no significant correlation was found for another related SNP (rs16991615) (218). Unfortunately, whether evidence for causal inference can be found using the entire GWAS summary statistics has not been addressed. Certain studies have suggested that the relationship between LM and the biological aging rate may be related to methylation in the blood, a marker of epigenetic aging (218), or regulated by the DDR pathway (219, 220), but the specific mechanism remains unclear and needs further investigation.

### 4.18 Other factors

In addition to the diseases and traits aforementioned, Magnus et al. also analyzed the causal relationship between ANM and liver function (alkaline phosphatase and alanine transaminase), kidney function (creatinine and urea), low density lipoprotein, and coeliac disease (103). However, a causal relationship was not found for any factor, except for coeliac disease based on the UKB GWAS. Nevertheless, these results were not as reliable as the negative results in the two-sample MR analysis as it was a one-sample MR analysis. Although the results demonstrated no correlation between ANM and these factors, some observational studies have found that postmenopausal women are more likely to develop hepatocellular carcinoma, non-alcoholic fatty liver disease, chronic kidney disease, and kidney stones (221–224). This is likely to be related to estrogen decline during menopause. Therefore, the causal relationship between ANM and other diseases, such as non-alcoholic fatty liver disease and kidney stones, remains to be evaluated by new MR studies with higher quality data.

## 5 ANM as a Mediator

### 5.1 From childhood obesity to breast cancer

A recent MR study found that ANM can mediate the protective effect of larger childhood body size on BC, and this effect is independent of adult body size (225). This finding is similar to certain observational studies and MR analyses (226–228). In addition, IGF-1, SHBG, testosterone, and AAM have also been found to be mediators of ANM (225). However, this MR study merely identified a number of potential mediating components and none of the evaluated traits were discovered to strongly mediate this impact on its own. It is plausible that some related traits may work together to contribute to the mediated impact, which should be investigated in multi-mediator MVMR analyses in future research.

### 5.2 From birthweight to breast cancer

Based on a principle of mediation analysis, Zhang et al. demonstrated that fetal-specific birthweight can indirectly affect BC risk in adulthood via the AAM and subsequently the ANM (229). This finding supported the findings of certain previous studies to an extent (230, 231) and questioned the findings of others (232, 233). It offers an unprecedented opportunity to determine the link between birthweight and BC. However, due to unavailability of relevant data, Zhang et al. did not further assess the dose-response association between birthweight and BC and the impact of birthweight on distinct subtypes of breast cancer. This area therefore needs further research.

## 6 Conclusion and future perspective

Traditional observational studies have found a number of connections between ANM and other diseases or traits. Confounding factors, reverse causation, and other potential biases, however, make it difficult to draw a causal relationship from these relationships. Due to its effectiveness in minimizing reverse causality and confounding variables as well as its ability to evaluate some time- and money-consuming factors, MR has gradually grown in importance as a tool in epidemiological research. Since 2012, several GWAS have successively identified susceptibility loci associated with ANM, and have identified previously unpredicted genes and pathways, such as genes implicated in DDR, immune function and mitochondrial biogenesis (42, 43, 216, 234–237). A recent GWAS in over 200,000 women of European ancestry has identified 290 ANM loci and also evaluated ANM loci of approximately 78,000 women of East Asian ancestry (43). Utilizing SNPs as an IV to examine the relationship between exposure and results is the key to MR analysis. As a result, even when investigating the same exposure or outcome, the results of an MR analysis may differ when different SNPs are included, which may account for the inconsistent results stated above.

**Figure 4:**
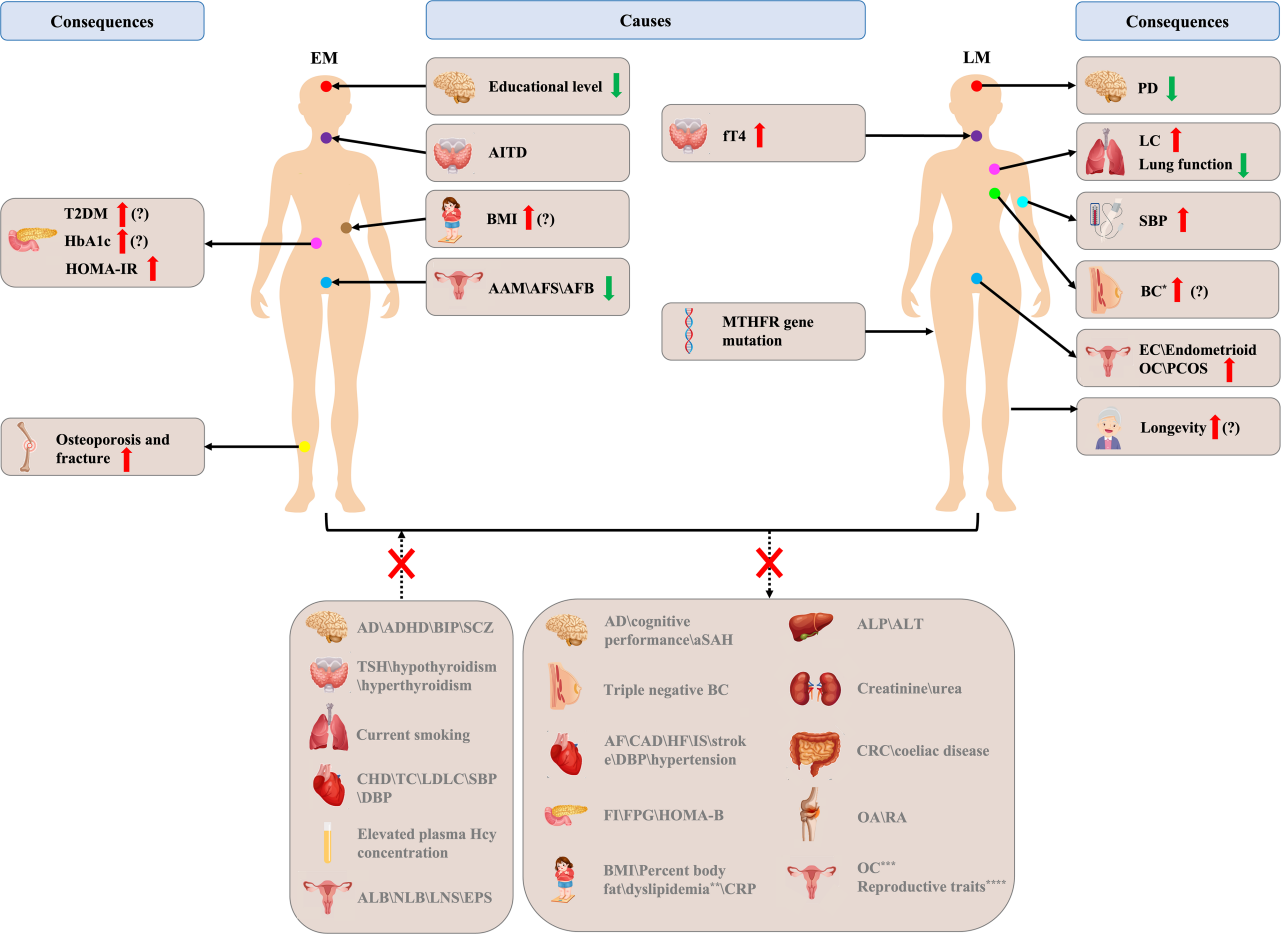
Brief overview of Mendelian randomization (MR) studies in age at natural menopause. EM, early menopause; LM, late menopause; T2DM, type 2 diabetes mellitus; HbA1c, glycosylated hemoglobin A1c; HOMA-IR, homeostasis model of insulin resistance; AITD, autoimmune thyroid disease; BMI, body mass index; AAM, age at menarche; AFS, age first had sexual intercourse; AFB, age at first birth; fT4, free thyroxine 4; MTHFR, methylene tetrahydrofolate reductase; PD, Parkinson’s disease; LC, lung cancer; SBP, systolic blood pressure; BC, breast cancer; EC, endometrial cancer; OC, ovarian cancer; AD, Alzheimer’s disease; ADHD, attention-deficit/hyperactivity disorder; BIP, bipolar disorder; SCZ, schizophrenia; TSH, thyroid stimulating hormone; CHD, coronary heart disease; TC, total cholesterol; LDLC, low-density lipoprotein cholesterol; DBP, diastolic blood pressure; ALB, age at last birth; NLB, number of live births; LNS, lifetime number of sexual partners; EPS, ever being parous; aSAH, aneurysmal subarachnoid hemorrhage; AF, atrial fibrillation; CAD, coronary artery disease; HF, heart failure; IS, ischemic stroke; FI, fasting insulin; FPG, fasting plasma glucose; HOMA-B, homeostasis model of B-cell function; CRP, C reactive protein; ALP, alkaline phosphatase; ALT, alanine transaminase; CRC, colorectal cancer; OA, osteoarthritis; RA, rheumatoid arthritis; The red up arrow indicates a higher incidence or a higher degree, and green down arrow the opposite; the question mark indicates that there are still inconsistencies in MR studies; ^*^ indicates ER+ BC ()?\ER- BC ()?\luminal A-like BC\luminal B-like BC\HER2-enriched-like BC; ^**^ indicates abnormal level of total cholesterol\high-density lipoprotein cholesterol\low-density lipoprotein cholesterol\triglycerides\apolipoprotein A1\apolipoprotein B; ^***^ indicates invasive epithelial OC, high grade serous OC, low grade serous OC, clear cell OC, low malignant potential OC; ^****^ indicates AAM\AFS\AFB\ALB\NLB\LNS\EPS.

Numerous MR studies have been conducted to clarify the causality between ANM and other traits and diseases. As shown in **Figure 4**, MR studies have suggested that poor educational level, higher BMI, early AAM, early AFB, early AFS, and AITD appear to play a causal role in EM etiology. Higher fT4 level and *MTHFR* gene mutation appear to be involved in LM etiology. Furthermore, EM has been found to be causally associated with an increased risk of osteoporosis, fracture, T2DM, HbA1c, and HOMA-IR, and LM has been found to be causally associated with an increased SBP, higher risk of BC, EC, endometrioid OC, and LC, airflow obstruction, PCOS, longevity, and a lower risk of PD. In addition, ANM is also a mediator for breast cancer caused by birth weight and childhood body size. However, studies on BMI, diabetes, BC, and longevity continue to have inconsistent results, which needs further exploration. In addition, genetically predicted ANM was not causally associated with current smoking status, dyslipidemia, lifetime number of sexual partners, ever being parous, number of live births, age at last birth, TSH, hypothyroidism, hyperthyroidism, elevated plasma Hcy concentration, psychiatric disorders, atrial fibrillation, coronary artery disease, heart failure, ischemic stroke, stroke, hypertension, percentage body fat, dyslipidemia, C reactive protein, triple negative BC, invasive epithelial OC, high grade serous OC, low grade serous OC, clear cell OC, low malignant potential OC, CRC, FVC, spirometric restriction, OA, AD, aSAH, OA, RA, fasting glucose, fasting insulin, homeostasis model of B-cell function, alkaline phosphatase, alanine transaminase, creatinine, urea, and coeliac disease. According to the research that has been published to date, the duration of estrogen exposure is a significant cause of these diseases or traits, but most of the specific underlying mechanisms remain unclear. Some sex hormones with large fluctuations during menopause transition, such as SHBG and bioavailable testosterone, may also be involved in menopausal related diseases. MR studies have validated previous observational studies with inconsistent results, whilst providing support for studies with the same results, questioning studies with contradictory results, and providing new directions for future research.

However, it is undeniable that MR research also has limitations. Firstly, as it is challenging to completely rule out pleiotropy, MR does not conclusively prove or disprove causation between an exposure and an outcome, despite being free of reverse causation and confounding factors that affect observational studies. To mitigate this, many MR studies conduct sensitivity analyses, which involve the use of methods like MR-Egger to detect pleiotropy and/or the exclusion of instrument SNPs with established relationships with possible confounders. When repeated MR analyses in distinct cohorts provide similar findings, a compelling case for causality (or absence of causality) can be made. Of the 31 included studies, sensitivity analysis was not performed in only 4 studies. In addition, some studies have verified the results of 1-2 replication cohorts. Therefore, the results of these MR analyses are highly credible. Secondly, it should be noted that the indicated causal relationships have been mostly found in cohorts of European descent and ANM-related SNPs only account for a relatively modest percentage of the estimated heritability of ANM. Thus, these results cannot be extrapolated to other racial/ethnic populations as a result. A way to counter this limitation while also increasing confidence to an MR result is to conduct a trans-ancestry study whereby the same exposure and outcome are tested in different populations using population-specific GWAS data. Unfortunately, although Ruth et al. (43) evaluated 290 ANM loci in approximately 78,000 women of East Asian ancestry, no relevant MR studies on ANM in women of East Asian ancestry have been reported due to substantial heterogeneity of effect sizes and allele frequencies. Thirdly, the data used were based on researcher self-report, which could lead to reporter recall bias and lower statistical power. Fourthly, it is impossible to deeply explore the role of ANM in the development of these diseases or traits due to the inability to stratify ANM (premature, early, and late), diseases (mild, moderate, and severe), and cancer stages due to the lack of epidemiological data. A GWAS on EM, however, failed to identify any novel genetic variations and demonstrated that EM and ANM have a common genetic origin. Therefore, the same polygenic variants as ANM also account for EM, at least in part (238).

Given the advantages of MR over observational studies, investigators can explore the risk factors and the consequences of abnormal ANM in the future using the results of large consortia that measure various traits, particularly in cases where previous studies produced ambiguous or contradictory results. In addition, more in-depth and rigorous experiments, such as stratification analysis, are needed to explain the inconsistencies found (e.g., in breast cancer and T2DM), and more MR studies on ANM risk factors (e.g., diet, vitamin D and calcium intake, and alcohol consumption) and outcomes (e.g., kidney stones and chronic kidney disease) should be on the agenda. The results of MR studies will aid us in improving the understanding of the development of abnormal reproductive traits and counseling patients, with the ultimate goal of preventing abnormal ANM in women at high risk and preventing adverse consequences in those who already have abnormal ANM.

## Author contributions

XZ, ZH, and SW contributed to the study design. XZ and ZH collected and analyzed all data. XZ wrote the manuscript, and SW revised the manuscript. All authors contributed to the article and approved the submitted version.
