# Supplementary material for: Review of mendelian randomization studies on age at natural menopause
Source: Front Endocrinol (Lausanne). 2023 Sep 11;14:1234324. doi: 10.3389/fendo.2023.1234324 (PMC10520463; doi:10.3389/fendo.2023.1234324)
Supplement: Supplementary file 1 [file Table_1.docx]

Supplementary Material

**Review of Mendelian Randomization Studies on Age at Natural Menopause**

**Xiao Zhang, Zhao Huangfu, Shaowei Wang^*^**

**^*^Correspondence:** Shaowei Wang: [w_sw999@163.com](mailto:w_sw999@163.com)

Supplementary Table 1 Mendelian randomization studies of various exposure with ANM as the outcome

| Exposure | Population | Cohorts | Sample size | Outcome | Population | Cohorts | Sample size | SNPs, n | MR methods | OR/B (95%CI) | P | Sensitivity tests | Reference |
| --- | --- | --- | --- | --- | --- | --- | --- | --- | --- | --- | --- | --- | --- |
| Lower BMI | European | UKB | 193570 | ANM | European | UKB | 111593 | 110 | IVW | -0.05 | 0.027 | Yes | (1) |
| BMI | European | GIANT | 339224 | ANM | European | ReproGen | 70000 | 56 | IVW | -0.069 (-0.294 to 0.156) | P>0.05 | No | (2) |
| Early AAM | European | ReproGen | 182416 | ANM | European | ReproGen | 69360 | 61 | IVW | -0.23 | 0.001 | Yes | (1) |
| Early AAM | European | UKB | 188644 | ANM | European | UKB | 111593 | 111 | IVW | 0.34 | 0.035 | Yes | (1) |
| AAM | European | UKB | 243898 | ANM | European | UKB | 143791 | 223 | IVW | 0.064 | 0.001 | Yes | (3) |
| AAM | European | UKB | 243898 | ANM | European | ReproGen (2015) | 69360 | 166 | IVW | 0.052 | 0.014 | Yes | (3) |
| AAM | European | UKB | 243898 | ANM | European | ReproGen (2021) | 201323 | 223 | IVW | 0.064 | 0.001 | Yes | (3) |
| AAM | European | ReproGen | 182416 | ANM | European | UKB | 143791 | 60 | IVW | 0.075 | 0.001 | Yes | (3) |
| AFB | European | UKB | 203606 | ANM | European | UKB | 143791 | 41 | IVW | 0.212 | 8E-8 | Yes | (3) |
| AFB | European | UKB | 203606 | ANM | European | ReproGen (2015) | 69360 | 33 | IVW | 0.014 | 0.824 | Yes | (3) |
| AFB | European | UKB | 203606 | ANM | European | ReproGen (2021) | 201323 | 40 | IVW | 0.075 | 0.098 | Yes | (3) |
| ALB | European | UKB | 203356 | ANM | European | UKB | 143791 | 9 | IVW | 0.149 | 0.101 | Yes | (3) |
| NLB | European | UKB | 250746 | ANM | European | UKB | 143791 | 9 | IVW | -0.057 | 0.677 | Yes | (3) |
| AFS | European | UKB | 219486 | ANM | European | UKB | 143791 | 53 | IVW | 0.110 | 0.003 | Yes | (3) |
| AFS | European | UKB | 219486 | ANM | European | ReproGen (2015) | 69360 | 39 | IVW | -0.007 | 0.895 | Yes | (3) |
| AFS | European | UKB | 219486 | ANM | European | ReproGen (2021) | 201323 | 53 | IVW | 0.044 | 0.212 | Yes | (3) |
| LNS | European | UKB | 208274 | ANM | European | UKB | 143791 | 34 | IVW | 0.065 | 0.210 | Yes | (3) |
| EPS | European | UKB | 250746 | ANM | European | UKB | 143791 | 4 | IVW | -0.675 | 0.108 | Yes | (3) |
| Poor educational level | European | SSGAC | 182286 | ANM | European | UKB | 111593 | 16 | IVW | 1.19 | 0.004 | Yes | (1) |
| Current smoking | European | UKB | 193956 | ANM | European | UKB | 111593 | 2 | IVW | 0.26 | >0.05 | Yes | (1) |
| TSH | European | ThyroidOmics | 54288 | ANM | European | ReproGen | 132989 | 27 | IVW | 0.001 (-0.008 to 0.010) | 0.832 | Yes | (4) |
| TSH | European | ThyroidOmics | 54288 | ANM | European | UKB | 111593 | 60 | IVW | -0.085 (-0.209 to 0.039) | 0.180 | Yes | (4) |
| TSH | European | ThyroidOmics | 54288 | ANM | European | UKB | 111593 | 60 | Standardized IVW | -0.017 (-0.043 to 0.008) | 0.173 | Yes | (4) |
| TSH restricted to AITD | European | ThyroidOmics | 54288 | ANM | European | ReproGen | 132989 | 10 | IVW | -0.009 (-0.009 to 0.027) | 0.311 | Yes | (4) |
| TSH restricted to AITD | European | ThyroidOmics | 54288 | ANM | European | UKB | 111593 | 15 | IVW | -0.395 (-0.139 to -0.650) | 0.003 | Yes | (4) |
| TSH restricted to AITD | European | ThyroidOmics | 54288 | ANM | European | UKB | 111593 | 15 | Standardized IVW | -0.079 (-0.023 to -0.134) | 0.005 | Yes | (4) |
| TSH restricted to no-AITD | European | ThyroidOmics | 54288 | ANM | European | ReproGen | 132989 | 17 | IVW | 0.008 (-0.003 to -0.020) | 0.157 | Yes | (4) |
| TSH restricted to no-AITD | European | ThyroidOmics | 54288 | ANM | European | UKB | 111593 | 43 | IVW | 0.024 (-0.108 to 0.157) | 0..721 | Yes | (4) |
| TSH restricted to no-AITD | European | ThyroidOmics | 54288 | ANM | European | UKB | 111593 | 43 | Standardized IVW | 0.004 (-0.023 to 0.030) | 0.790 | Yes | (4) |
| fT4 | European | ThyroidOmics | 49269 | ANM | European | ReproGen | 132989 | 14 | IVW | -0.020 (-0.000 to -0.039) | 0.046 | Yes | (4) |
| fT4 | European | ThyroidOmics | 49269 | ANM | European | UKB | 111593 | 31 | IVW | 0.151 (-0.052 to 0.354) | 0.146 | Yes | (4) |
| fT4 | European | ThyroidOmics | 49269 | ANM | European | UKB | 111593 | 31 | Standardized IVW | 0.032 (-0.012 to 0.075) | 0.152 | Yes | (4) |
| fT4 restricted to DIO1+DIO2 genes | European | ThyroidOmics | 49269 | ANM | European | ReproGen | 132989 | 5 | IVW | -0.018 (0.009 to -0.046) | 0.194 | Yes | (4) |
| fT4 restricted to DIO1+DIO2 genes | European | ThyroidOmics | 49269 | ANM | European | UKB | 111593 | 7 | IVW | -0.101 (-0.322 to 0.120) | 0.372 | Yes | (4) |
| fT4 restricted to DIO1+DIO2 genes | European | ThyroidOmics | 49269 | ANM | European | UKB | 111593 | 7 | Standardized IVW | -0.029 (-0.072 to 0.014) | 0.182 | Yes | (4) |
| fT4 restricted to no-DIO1+DIO2 genes | European | ThyroidOmics | 49269 | ANM | European | ReproGen | 132989 | 9 | IVW | -0.021 (-0.009 to 0.050) | 0.176 | Yes | (4) |
| fT4 restricted to no-DIO1+DIO2 genes | European | ThyroidOmics | 49269 | ANM | European | UKB | 111593 | 24 | IVW | 0.288 (0.026 to 0.551) | 0.031 | Yes | (4) |
| fT4 restricted to no-DIO1+DIO2 genes | European | ThyroidOmics | 49269 | ANM | European | UKB | 111593 | 24 | Standardized IVW | 0.065 (0.010 to 0.120) | 0.020 | Yes | (4) |
| Subclinical hypothyroidism | European | ThyroidOmics | 53241 | ANM | European | ReproGen | 132989 | 5 | IVW | -0.000 (-0.007 to 0.008) | 0.918 | Yes | (4) |
| Subclinical hypothyroidism | European | ThyroidOmics | 53241 | ANM | European | UKB | 111593 | 8 | IVW | -0.013 (-0.138 to 0.113) | 0.843 | Yes | (4) |
| Subclinical hypothyroidism | European | ThyroidOmics | 53241 | ANM | European | UKB | 111593 | 8 | Standardized IVW | -0.006 (-0.032 to 0.019) | 0.624 | Yes | (4) |
| Overt hypothyroidism | European | 23andMe | 134641 | ANM | European | ReproGen | 132989 | 10 | IVW | 0.001 (-0.009 to 0.010) | 0.876 | Yes | (4) |
| Overt hypothyroidism | European | 23andMe | 134641 | ANM | European | UKB | 111593 | 18 | IVW | -0.019 (-0.129 to 0.092) | 0.738 | Yes | (4) |
| Overt hypothyroidism | European | 23andMe | 134641 | ANM | European | UKB | 111593 | 18 | Standardized IVW | -0.007 (-0.029 to 0.016) | 0.571 | Yes | (4) |
| Subclinical hyperthyroidism | European | ThyroidOmics | 51668 | ANM | European | ReproGen | 132989 | 6 | IVW | 0.001 (-0.003 to 0.005) | 0.660 | Yes | (4) |
| Subclinical hyperthyroidism | European | ThyroidOmics | 51668 | ANM | European | UKB | 111593 | 8 | IVW | -0.008 (-0.098 to 0.083) | 0.869 | Yes | (4) |
| Subclinical hyperthyroidism | European | ThyroidOmics | 51668 | ANM | European | UKB | 111593 | 8 | Standardized IVW | -0.002 (-0.019 to 0.016) | 0.854 | Yes | (4) |
| Subclinical hyperthyroidism excluding FOXE1 | European | ThyroidOmics | 51668 | ANM | European | ReproGen | 132989 | 5 | IVW | 0.001 (-0.003 to 0.005) | 0.651 | Yes | (4) |
| Subclinical hyperthyroidism excluding FOXE1 | European | ThyroidOmics | 51668 | ANM | European | UKB | 111593 | 7 | IVW | -0.010 (-0.112 to 0.017) | 0.843 | Yes | (4) |
| Subclinical hyperthyroidism excluding FOXE1 | European | ThyroidOmics | 51668 | ANM | European | UKB | 111593 | 7 | Standardized IVW | -0.002 (-0.021 to 0.017) | 0.841 | Yes | (4) |
| Elevated plasma homocysteine concentrations | European | Large cohort (van et al.) | 44147 | ANM | European | ReproGen | 69360 | 18 | IVW | 0.13 (-0.06 to 0.32) | 0.180 | Yes | (5) |
| Elevated plasma homocysteine concentrations | European | Large cohort (van et al.) | 44147 | ANM | European | UKB | 111593 | 18 | IVW | 0.17 (-0.05 to 0.40) | 0.130 | Yes | (5) |
| Elevated plasma homocysteine concentrations | European | Large cohort (van et al.) | 44147 | ANM | European | UKB | 111593 | 18 | IVW | 0.03 (-0.01 to 0.08) | 0.150 | Yes | (5) |
| MTHFR rs1801133 | European | Large cohort (van et al.) | 44147 | ANM | European | UKB | 111593 | 1 | Ward ratio | 0.62 (0.34 to 0.90) | P<0.05 | Yes | (5) |
| MTHFR, MTR, CBS | European | Large cohort (van et al.) | 44147 | ANM | European | UKB | 111593 | 5 | IVW | 0.37 (-0.03 to 0.78) | P>0.05 | Yes | (5) |
| MTHFR, MTR, CBS, MMACHC, CUBN, FUT2, MUT | European | Large cohort (van et al.) | 44147 | ANM | European | UKB | 111593 | 10 | IVW | 0.24 (-0.08 to 0.57) | P>0.05 | Yes | (5) |
| CHD | European | m-CARDIoGRAMplusC4D | 185000 | ANM | European | ReproGen | 70000 | 56 | IVW | 0.063 (-0.050 to 0.176) | P>0.05 | No | (2) |
| Total cholesterol | European | GLGC | 188577 | ANM | European | ReproGen | 70000 | 56 | IVW | -0.002 (-0.006 to 0.002) | P>0.05 | No | (2) |
| LDL cholesterol | European | GLGC | 188577 | ANM | European | ReproGen | 70000 | 56 | IVW | 0.003 (-0.001 to 0.006) | P>0.05 | No | (2) |
| SBP | European | ICBP | 200000 | ANM | European | ReproGen | 70000 | 56 | IVW | 0.015 (-0.018 to 0.049) | P>0.05 | No | (2) |
| DBP | European | ICBP | 200000 | ANM | European | ReproGen | 70000 | 56 | IVW | 0.023 (-0.031 to 0.077) | P>0.05 | No | (2) |
| AD | European | IGAP | 54162 | ANM | European | ReproGen | 69360 | 6 | IVW | -0.044 (-0.206 to 0.119) | 0.598 | Yes | (6) |
| ADHD | European | PGC | 55374 | ANM | European | UKB | 102386 | - | IVW | -0.209 | 0.144 | Yes | (7) |
| BIP | European | PGC | 16731 | ANM | European | UKB | 102386 | - | IVW | -0.137 | 0.197 | Yes | (7) |
| SCZ | European | PGC | 150064 | ANM | European | UKB | 102386 | - | IVW | 0.001 | 0.974 | Yes | (7) |

Note: SNP, single nucleotide polymorphisms; MR, mendelian randomization; OR, odds ratio; BMI, body mass index; UKB, UK Biobank; ANM, age at menopause; IVW, inverse-variance weighted; AAM, age at menarche; SSGAC, Social Science Genetic Association Consortium; AFB, age at first live birth; ALB, age at last live birth; NLB, number of live births; AFS, age at first sexual intercourse; LNS, lifetime number of sexual partners; EPS, ever parous status; TSH, thyroid stimulating hormone; AITD, autoimmune thyroid disease; fT4, free thyroxine 4; MTHFR, methylene tetrahydrofolate reductase; MTR, 5-methyltetrahydrofolate homocysteine methyltransferase; CBS, cystathionine B synthase; MMACHC, methylmalonix acidemia is associated with a C-protein associated gene for homocysteinemia; CUBN, mutations in either cubilin; FUT2, fucosyltransferase 2; MUT, methylmalonyl-coa mutase defective type; CHD, coronary heart disease; GLGC, global lipids genetics consortium; LDL, low-density lipoprotein; SBP, systolic blood pressure; ICBP, international consortium of blood pressure genome wide association studies; DBP, diastolic blood pressure; GIANT, genetic investigation of anthropometric traits; AD, Alzheimer’s disease; IGAP, international genomics of Alzheimer’s project; ADHD, attention-deficit/hyperactivity disorder; PGC, psychiatric genomics consortium; BIP, bipolar disorder; SCZ, schizophrenia.

**References**

1. Ding, X., Tang, R., Zhu, J., He, M., Huang, H., Lin, Z., et al. An Appraisal of the Role of Previously Reported Risk Factors in the Age at Menopause Using Mendelian Randomization. *Front Genet* (2020) 11(507. doi: 10.3389/fgene.2020.00507.

2. Ardissino, M., Slob, E.A.W., Carter, P., Rogne, T., Girling, J., Burgess, S., et al. Sex-Specific Reproductive Factors Augment Cardiovascular Disease Risk in Women: A Mendelian Randomization Study. *J Am Heart Assoc* (2023) 12(5):e027933. doi: 10.1161/jaha.122.027933.

3. Prince, C., Sharp, G.C., Howe, L.D., Fraser, A., Richmond, R.C. The relationships between women's reproductive factors: a Mendelian randomisation analysis. *BMC Med* (2022) 20(1):103. doi: 10.1186/s12916-022-02293-5.

4. Kjaergaard, A.D., Marouli, E., Papadopoulou, A., Deloukas, P., Kuś, A., Sterenborg, R., et al. Thyroid function, sex hormones and sexual function: a Mendelian randomization study. *Eur J Epidemiol* (2021) 36(3):335-44. doi: 10.1007/s10654-021-00721-z.

5. Kjaergaard, A.D., Wu, Y., Ming, W.K., Wang, Z., Kjaergaard, M.N., Ellervik, C. Homocysteine and female fertility, pregnancy loss and offspring birthweight: a two-sample Mendelian randomization study. *Eur J Clin Nutr* (2022) 76(1):40-7. doi: 10.1038/s41430-021-00898-2.

6. Li, M., Lin, J., Liang, S., Chen, Z., Bai, Y., Long, X., et al. The role of age at menarche and age at menopause in Alzheimer's disease: evidence from a bidirectional mendelian randomization study. *Aging (Albany NY)* (2021) 13(15):19722-49. doi: 10.18632/aging.203384.

7. Ni, G., Amare, A.T., Zhou, X., Mills, N., Gratten, J., Lee, S.H. The genetic relationship between female reproductive traits and six psychiatric disorders. *Sci Rep* (2019) 9(1):12041. doi: 10.1038/s41598-019-48403-x.
