# Supplementary material for: Review of mendelian randomization studies on age at natural menopause
Source: Front Endocrinol (Lausanne). 2023 Sep 11;14:1234324. doi: 10.3389/fendo.2023.1234324 (PMC10520463; doi:10.3389/fendo.2023.1234324)
Supplement: Supplementary file 2 [file Table_2.docx]

Supplementary Material

**Review of Mendelian Randomization Studies on Age at Natural Menopause**

**Xiao Zhang, Zhao Huangfu, Shaowei Wang^*^**

**^*^Correspondence:** Shaowei Wang: [w_sw999@163.com](mailto:w_sw999@163.com)

Supplementary Table 2 Mendelian randomization studies of various outcomes where ANM is the exposure.

| Exposure | Population | Cohorts | Sample size | Outcome | Population | Cohorts | Sample size | SNPs, n | MR methods | OR/B (95%CI) | P | Sensitivity tests | Reference |
| --- | --- | --- | --- | --- | --- | --- | --- | --- | --- | --- | --- | --- | --- |
| ANM | European | ReproGen (2021) | 201323 | AF | European | HUNT, deCODE, MGI, DiscovEHR, UKB, AFGen | 1030836 | 154 | IVW | 1.00 (0.99 to 1.01) | 0.94 | Yes | (1) |
| ANM | European | ReproGen (2021) | 201323 | AF | European | UKB | 208235 | - | IVW | 1.01 (0.99 to 1.03) | 0.337 | Yes | (1) |
| ANM | European | ReproGen (2021) | 201323 | CAD | European | UKB and CARDloGRAMplusC4D | 547261 | 141 | IVW | 1.00 (0.99 to 1.01) | 0.894 | Yes | (1) |
| ANM | European | ReproGen (2021) | 201323 | CAD | European | UKB | 210617 | - | IVW | 1.01 (0.99 to 1.03) | 0.599 | Yes | (1) |
| ANM | European | ReproGen (2021) | 201323 | CAD | European | ReproGen (2021) | 201323 | 227 | IVW | 1.002 (0.992 to 1.013) | 0.65 | Yes | (2) |
| ANM | European | ReproGen (2015) | 70000 | CHD | European | UKB, m-CARDIoGRAMplusC4D, EPIC-CVD | 417579 | 56 | IVW | 0.99 (0.97 to 1.01) | P>0.05 | No | (3) |
| ANM | European | WHI | 17357 | CHD | European | WHI | 17357 | - | One-sample MR | 0.83 (0.61 to 1.13) | P>0.05 | Yes | (4) |
| ANM | European | UKB | 94074 | CHD | European | UKB | 94074 | - | One-sample MR | 0.94 (0.74 to 1.19) | P>0.05 | Yes | (4) |
| ANM | European | CHS | 1902 | CHD | European | CHS | 1902 | - | One-sample MR | 1.25 (0.68 to 2.31) | P>0.05 | Yes | (4) |
| ANM | European | ARIC | 4872 | CHD | European | ARIC | 4872 | - | One-sample MR | 1.04 (0.57 to 1.86) | P>0.05 | Yes | (4) |
| ANM | European | FHS | 1294 | CHD | European | FHS | 1294 | - | One-sample MR | 0.55 (0.16 to 1.84) | P>0.05 | Yes | (4) |
| ANM | European | MESA | 1089 | CHD | European | MESA | 1089 | - | One-sample MR | 2.15 (0.42 to 10.90) | P>0.05 | Yes | (4) |
| ANM | European | WHI+UKB | 111431 | CHD | European | WHI+UKB | 111431 | - | One-sample MR | 0.90 (0.74 to 1.08) | P>0.05 | Yes | (4) |
| ANM | European | All | 120588 | CHD | European | All | 120588 | - | One-sample MR | 0.93 (0.79 to 1.10) | P>0.05 | Yes | (4) |
| ANM | European | ReproGen (2021) | 201323 | HF | European | HERMES | 977323 | 152 | IVW | 1.00 (0.99 to1.01) | 0.735 | Yes | (1) |
| ANM | European | ReproGen (2021) | 201323 | HF | European | UKB | 202943 | - | IVW | 1.01 (0.98 to 1.04) | 0.651 | Yes | (1) |
| ANM | European | ReproGen (2021) | 201323 | IS | European | MEGASTROKE | 440328 | 151 | IVW | 1.00 (0.98 to 1.01) | 0.693 | Yes | (1) |
| ANM | European | ReproGen (2021) | 201323 | IS | European | UKB | 201592 | - | IVW | 1.01 (0.97 to 1.04) | 0.663 | Yes | (1) |
| ANM | European | ReproGen (2021) | 201323 | IS | European | MEGASTROKE | 440328 | 218 | IVW | 1.00 (0.98 to 1.01) | 0.657 | Yes | (5) |
| ANM controlled for EA and BMI | European | ReproGen (2021) | 201323 | IS | European | MEGASTROKE | 440328 | 479 | IVW | 1.00 (0.99 to 1.02) | 0.874 | Yes | (5) |
| ANM | European | WHI | 17723 | IS | European | WHI | 17723 | - | One-sample MR | 0.58 (0.40 to 0.86) | P<0.05 | Yes | (4) |
| ANM | European | UKB | 95273 | IS | European | UKB | 95273 | - | One-sample MR | 1.27 (0.94 to 1.70) | P>0.05 | Yes | (4) |
| ANM | European | CHS | 1925 | IS | European | CHS | 1925 | - | One-sample MR | 1.28 (0.55 to 2.96) | P>0.05 | Yes | (4) |
| ANM | European | ARIC | 4878 | IS | European | ARIC | 4878 | - | One-sample MR | 0.63 (0.24 to 1.68) | P>0.05 | Yes | (4) |
| ANM | European | WTCCC2 | 3193 | IS | European | WTCCC2 | 3193 | - | One-sample MR | 0.66 (0.29 to 1.49) | P>0.05 | Yes | (4) |
| ANM | European | FHS | 1303 | IS | European | FHS | 1303 | - | One-sample MR | 0.69 (0.10 to 4.61) | P>0.05 | Yes | (4) |
| ANM | European | MESA | 1089 | IS | European | MESA | 1089 | - | One-sample MR | 1.02 (0.08 to 13.25) | P>0.05 | Yes | (4) |
| ANM | European | WHI+UKB | 112996 | IS | European | WHI+UKB | 112996 | - | One-sample MR | 0.87 (0.41 to 1.86) | P>0.05 | Yes | (4) |
| ANM | European | All | 125384 | IS | European | All | 125384 | - | One-sample MR | 0.85 (0.58 to 1.25) | P>0.05 | Yes | (4) |
| ANM | European | ReproGen (2021) | 201323 | stroke | European | MEGASTROKE | 446696 | 151 | IVW | 1.00 (0.98 to 1.01) | 0.606 | Yes | (1) |
| ANM | European | ReproGen (2021) | 201323 | stroke | European | UKB | 204226 | - | IVW | 1.00 (0.98 to 1.03) | 0.892 | Yes | (1) |
| ANM | European | ReproGen (2021) | 201323 | Small vessel stroke | European | MEGASTROKE | 411497 | 218 | IVW | 1.00 (0.97 to 1.03) | 0.86 | Yes | (5) |
| ANM controlled for EA and BMI | European | ReproGen (2021) | 201323 | Small vessel stroke | European | MEGASTROKE | 411497 | 479 | IVW | 1.00 (0.96 to 1.03) | 0.835 | Yes | (5) |
| ANM | European | ReproGen (2021) | 201323 | Large-artery atherosclerotic stroke | European | MEGASTROKE | 410484 | 218 | IVW | 0.99 (0.95 to 1.02) | 0.488 | Yes | (5) |
| ANM controlled for EA and BMI | European | ReproGen (2021) | 201323 | Large-artery atherosclerotic stroke | European | MEGASTROKE | 410484 | 479 | IVW | 0.99 (0.96 to 1.03) | 0.761 | Yes | (5) |
| ANM | European | ReproGen (2021) | 201323 | Cardioembolic stroke | European | MEGASTROKE | 413304 | 218 | IVW | 1.02 (0.99 to 1.05) | 0.06 | Yes | (5) |
| ANM controlled for EA and BMI | European | ReproGen (2021) | 201323 | Cardioembolic stroke | European | MEGASTROKE | 413304 | 479 | IVW | 1.02 (0.99 to 1.05) | 0.121 | Yes | (5) |
| ANM (RS-I-3-crude) | European | Rotterdam Study cohort | 1603 | SBP | European | UKB | 168575 | - | One-sample MR | -1.89 (-4.23 to 0.45) | 0.11 | - | (6) |
| ANM (RS-I-3 adjusting for antihypertensive medication) | European | Rotterdam Study cohort | 1603 | SBP | European | UKB | 168576 | - | One-sample MR | -1.86 (-4.13 to 0.41) | 0.11 | - | (6) |
| ANM (RS-II-1-crude) | European | Rotterdam Study cohort | 790 | SBP | European | UKB | 168578 | - | One-sample MR | 0.68 (-2.50 to 3.87) | 0.68 | - | (6) |
| ANM (RS-II-1-adjusting for antihypertensive medication) | European | Rotterdam Study cohort | 790 | SBP | European | UKB | 168579 | - | One-sample MR | -0.76 (-3.92 to 2.38) | 0.63 | - | (6) |
| ANM (RS-III-1-crude) | European | Rotterdam Study cohort | 919 | SBP | European | UKB | 168581 | - | One-sample MR | 0.93 (-1.50 to 3.38) | 0.45 | - | (6) |
| ANM (RS-III-1-adjusting for antihypertensive medication) | European | Rotterdam Study cohort | 919 | SBP | European | UKB | 168582 | - | One-sample MR | 0.40 (2.06 to 2.87) | 0.75 | - | (6) |
| ANM (Colaus-crude) | European | Colaus study | 1139 | SBP | European | UKB | 168575 | - | One-sample MR | -0.80 (-4.17 to 2.61) | 0.65 | - | (6) |
| ANM (Colaus- adjusting for antihypertensive medication) | European | Colaus study | 1139 | SBP | European | UKB | 168575 | - | One-sample MR | -0.84 (-3.72 to 2.05) | 0.57 | - | (6) |
| ANM (crude) | European | Rotterdam Study cohort and Colaus study | 4451 | SBP | European | UKB | 168575 | - | One-sample MR | -0.36 (-1.72 to 1.01) | P>0.05 | - | (6) |
| ANM (adjusting for antihypertensive medication) | European | Rotterdam Study cohort and Colaus study | 4451 | SBP | European | UKB | 168576 | - | One-sample MR | -0.82 (-2.13 to 0.50) | P>0.05 | - | (6) |
| ANM excluded due to LD with other variants | European | ReproGen (2015) | 69360 | SBP | European | UKB | 168575 | 40 | IVW | 0.25 (0.04 to 0.45) | 0.02 | Yes | (6) |
| ANM restricted DDR genes | European | ReproGen (2015) | 69360 | SBP | European | UKB | 168575 | 29 | IVW | 0.291 | 0.0151 | Yes | (6) |
| ANM restricted non-DDR genes | European | ReproGen (2015) | 69360 | SBP | European | UKB | 168575 | 17 | IVW | 0.175 | 0.389 | Yes | (6) |
| ANM excluded SNPs associated with BMI | European | ReproGen (2015) | 69360 | SBP | European | UKB | 168575 | 37 | IVW | 0.252 | 0.021 | Yes | (6) |
| ANM excluded SNPs associated with menarche | European | ReproGen (2015) | 69360 | SBP | European | UKB | 168575 | 38 | IVW | 0.277 | 0.01 | Yes | (6) |
| ANM | European | ReproGen (2015) | 69360 | SBP | European | UKB | 168575 | 53 | IVW | 0.209 | 0.015 | Yes | (6) |
| ANM (RS-I-3-crude) | European | Rotterdam Study cohort | 1603 | DBP | European | UKB | 168577 | - | One-sample MR | -0.57 (-1.72 to 0.58) | 0.33 | - | (6) |
| ANM (RS-I-3 adjusting for antihypertensive medication) | European | Rotterdam Study cohort | 1603 | DBP | European | UKB | 168578 | - | One-sample MR | -0.56 (-1.68 to 0.56) | 0.33 |  | (6) |
| ANM (RS-II-1-crude) | European | Rotterdam Study cohort | 790 | DBP | European | UKB | 168580 | - | One-sample MR | 0.48 (-1.06 to 2.02) | 0.54 | - | (6) |
| ANM (RS-II-1-adjusting for antihypertensive medication) | European | Rotterdam Study cohort | 790 | DBP | European | UKB | 168581 | - | One-sample MR | 0.27 (-1.42 to 1.98) | 0.75 |  | (6) |
| ANM (RS-III-1-crude) | European | Rotterdam Study cohort | 919 | DBP | European | UKB | 168583 | - | One-sample MR | 0.41 (-0.95 to 1.77) | 0.56 | - | (6) |
| ANM (RS-III-1-adjusting for antihypertensive medication) | European | Rotterdam Study cohort | 919 | DBP | European | UKB | 168584 | - | One-sample MR | 0.10 (-1.29 to 1.48) | 0.89 |  | (6) |
| ANM (Colaus-crude) | European | Colaus study | 1139 | DBP | European | UKB | 168576 | - | One-sample MR | -0.02 (-1.85 to 1.81) | 0.98 | - | (6) |
| ANM (Colaus- adjusting for antihypertensive medication) | European | Colaus study | 1139 | DBP | European | UKB | 168577 | - | One-sample MR | 0.25 (-1.40 to 1.90) | 0.77 |  | (6) |
| ANM (crude) | European | Rotterdam Study cohort and Colaus study | 4451 | DBP | European | UKB | 168577 | - | One-sample MR | -0.01 (-0.71 to 0.70) | P>0.05 | - | (6) |
| ANM (adjusting for antihypertensive medication) | European | Rotterdam Study cohort and Colaus study | 4451 | DBP | European | UKB | 168578 | - | One-sample MR | -0.10 (-0.81 to 0.60) | P>0.05 |  | (6) |
| ANM excluded due to LD with other variants | European | ReproGen (2015) | 69360 | DBP | European | UKB | 168575 | 40 | IVW | 0.05 (-0.08 to 0.17) | 0.46 | Yes | (6) |
| ANM restricted DDR genes | European | ReproGen (2015) | 69360 | DBP | European | UKB | 168575 | 29 | IVW | 0.071 | 0.353 | Yes | (6) |
| ANM restricted non-DDR genes | European | ReproGen (2015) | 69360 | DBP | European | UKB | 168575 | 17 | IVW | -0.046 | 0.732 | Yes | (6) |
| ANM excluded SNPs associated with BMI | European | ReproGen (2015) | 69360 | DBP | European | UKB | 168575 | 37 | IVW | 0.052 | 0.439 | Yes | (6) |
| ANM excluded SNPs associated with menarche | European | ReproGen (2015) | 69360 | DBP | European | UKB | 168575 | 38 | IVW | 0.059 | 0.386 | Yes | (6) |
| ANM | European | ReproGen (2015) | 69360 | DBP | European | UKB | 168575 | 53 | IVW | 0.046 | 0.394 | Yes | (6) |
| ANM (RS-I-3-crude) | European | Rotterdam Study cohort | 1603 | Hypertension | European | UKB | 168579 | - | One-sample MR | 0.90 (0.71 to 1.13) | 0.35 | - | (6) |
| ANM (RS-I-3 adjusting for antihypertensive medication) | European | Rotterdam Study cohort | 1603 | Hypertension | European | UKB | 168577 | - | One-sample MR | 0.94 (0.73 to 1.43) | 0.63 | - | (6) |
| ANM (RS-II-1-crude) | European | Rotterdam Study cohort | 790 | Hypertension | European | UKB | 168582 | - | One-sample MR | 1.01 (0.81 to 1.47) | 0.55 | - | (6) |
| ANM (RS-II-1-adjusting for antihypertensive medication) | European | Rotterdam Study cohort | 790 | Hypertension | European | UKB | 168583 | - | One-sample MR | 1.02 (0.73 to 1.43) | 0.9 | - | (6) |
| ANM (RS-III-1-crude) | European | Rotterdam Study cohort | 919 | Hypertension | European | UKB | 168585 | - | One-sample MR | 0.97 (0.76 to 1.24) | 0.84 | - | (6) |
| ANM (RS-III-1-adjusting for antihypertensive medication) | European | Rotterdam Study cohort | 919 | Hypertension | European | UKB | 168583 | - | One-sample MR | 0.92 (0.71 to 1.21) | 0.56 | - | (6) |
| ANM (Colaus-crude) | European | Colaus study | 1139 | Hypertension | European | UKB | 168578 | - | One-sample MR | 1.09 (0.76 to 1.54) | 0.68 | - | (6) |
| ANM (Colaus- adjusting for antihypertensive medication) | European | Colaus study | 1139 | Hypertension | European | UKB | 168579 | - | One-sample MR | 1.03 (0.70 to 1.50) | 0.89 | - | (6) |
| ANM (crude) | European | Rotterdam Study cohort and Colaus study | 4451 | Hypertension | European | UKB | 168579 | - | One-sample MR | 0.97 (0.85 to 1.11) | P>0.05 | - | (6) |
| ANM (adjusting for antihypertensive medication) | European | Rotterdam Study cohort and Colaus study | 4451 | Hypertension | European | UKB | 168580 | - | One-sample MR | 0.96 (0.83 to 1.11) | P>0.05 | - | (6) |
| ANM | European | ReproGen (2021) | 201323 | T2DM | European | Large cohorts (Xue et al.) | 659316 | - | IVW | 1.01 (0.99 to 1.03) | P>0.05 | Yes | (7) |
| ANM | European | ReproGen (2021) | 201323 | T2DM | European | UKB | 181279 | - | One-sample MR | 1.07 (1.04 to 1.10) | P<0.05 | Yes | (7) |
| ANM | European | ReproGen (2021) | 201323 | T2DM | European | ReproGen (2021) | 201323 | 194 | IVW | 0.981 (0.970 to 0.992) | 1.1*10-3 | Yes | (2) |
| ANM | European | ReproGen (2015) | 69360 | T2DM | European | DIAGRAM | 464389 | - | IVW | 0.98 (0.96 to 1.01) | 0.7 | Yes | (8) |
| ANM without SNPs linked to BMI in childhood and adulthood | European | ReproGen (2015) | 69360 | T2DM | European | DIAGRAM | 464389 | - | IVW | 0.98 (0.95 to 1.01) | 0.32 | Yes | (8) |
| ANM | European | ReproGen (2015) | 69360 | FI | European | MAGIC | 51750 | 37 | IVW | -0.006 (-0.014 to 0.001) | 0.082 | Yes | (8) |
| ANM without SNPs linked to BMI in childhood and adulthood | European | ReproGen (2015) | 69360 | FI | European | MAGIC | 51750 | 33 | IVW | -0.007 (-0.016 to 0.002) | 0.115 | Yes | (8) |
| ANM | European | ReproGen (2015) | 69360 | FPG | European | MAGIC | 58074 | 36 | IVW | -0.007 (-0.016 to 0.002) | 0.118 | Yes | (8) |
| ANM without SNPs linked to BMI in childhood and adulthood | European | ReproGen (2015) | 69360 | FPG | European | MAGIC | 58074 | 33 | IVW | -0.006 (-0.015 to 0.004) | 0.256 | Yes | (8) |
| ANM | European | ReproGen (2015) | 69360 | HOMA-B | European | MAGIC | 36466 | 37 | IVW | -0.005 (-0.011 to 0.001) | 0.126 | Yes | (8) |
| ANM without SNPs linked to BMI in childhood and adulthood | European | ReproGen (2015) | 69360 | HOMA-B | European | MAGIC | 36466 | 33 | IVW | -0.004 (-0.011 to 0.003) | 0.303 | Yes | (8) |
| ANM | European | ReproGen (2015) | 69360 | HOMA-IR | European | MAGIC | 37037 | 37 | IVW | -0.011 (-0.019 to -0.004)] | 0.004 | Yes | (8) |
| ANM without SNPs linked to BMI in childhood and adulthood | European | ReproGen (2015) | 69360 | HOMA-IR | European | MAGIC | 37037 | 33 | IVW | -0.014 (-0.023 to -0.005) | 0.002 | Yes | (8) |
| ANM | European | ReproGen (2021) | 201323 | Fasting glucose | European | ReproGen (2021) | 201323 | 117 | IVW | 0.000 (-0.006 to 0.005) | 0.89 | Yes | (2) |
| ANM | European | ReproGen (2015) | 70000 | Fasting glucose | European | MAGIC | 122744 | 56 | IVW | -0.003 (-0.010 to 0.004) | P>0.05 | No | (3) |
| ANM | European | ReproGen (2021) | 201323 | Fasting insulin | European | ReproGen (2021) | 201323 | 127 | IVW | -0.004 (-0.01 to 0.002) | 0.16 | Yes | (2) |
| ANM | European | ReproGen (2015) | 70000 | HbA1c | European | MAGIC | 122744 | 56 | IVW | -0.014 (-0.030 to 0.002) | P>0.05 | No | (3) |
| ANM | European | ReproGen (2021) | 201323 | HbA1c | European | Large cohorts (Wheeler et al.) | 123665 | - | IVW | 0.0032 (0.0001 to 0.0054) | P<0.05 | Yes | (7) |
| ANM | European | ReproGen (2021) | 201323 | HbA1c | European | UKB | 181279 | - | One-sample MR | 0.0778 (0.0494 to 0.1063) | P<0.05 | Yes | (7) |
| ANM | European | ReproGen (2015) | 69360 | BMI | European | GIANT | 322154 | 36 | IVW | -0.003 (-0.010 to 0.004) | 0.365 | Yes | (9) |
| ANM | European | ReproGen (2021) | 201323 | BMI | European | ReproGen (2021) | 201323 | 210 | IVW | -0.003 (-0.008 to 0.003) | 0.35 | Yes | (2) |
| ANM | European | ReproGen (2021) | 201323 | Percent body fat | European | Large cohorts (Lu et al.) | 100716 | - | IVW | 0.0064 (-0.0020 to 0.0147) | P>0.05 | Yes | (7) |
| ANM | European | ReproGen (2021) | 201323 | Percent body fat | European | UKB | 181279 | - | One-sample MR | 0.0071 (-0.0257 to 0.0400) | P>0.05 | Yes | (7) |
| ANM | European | ReproGen (2015) | 70000 | Total cholesterol | European | GLGC | 188577 | 56 | IVW | -0.009 (-0.019 to 0.001) | P>0.05 | No | (3) |
| ANM | European | ReproGen (2015) | 70000 | HDL cholesterol | European | GLGC | 188577 | 56 | IVW | 0.000 (-0.004 to 0.003) | P>0.05 | No | (3) |
| ANM | European | ReproGen (2015) | 70000 | Triglycerides | European | GLGC | 188577 | 56 | IVW | 0.000 (-0.012 to 0.012) | P>0.05 | No | (3) |
| ANM | European | ReproGen (2021) | 201323 | LDL | European | GLGC 2013 | 188577 | - | IVW | 0.005 (-0.003 to 0.014) | P>0.05 | Yes | (7) |
| ANM | European | ReproGen (2021) | 201323 | LDL | European | UKB | 181279 | - | One-sample MR | -0.005 (-0.009 to -0.000) | P>0.05 | Yes | (7) |
| ANM | European | ReproGen (2015) | 70000 | ApoA1 | European | - | - | 56 | IVW | 0.000 (-0.002 to 0.003) | P>0.05 | No | (3) |
| ANM | European | ReproGen (2015) | 70000 | ApoB | European | - | - | 56 | IVW | -0.001 (-0.003 to 0.001) | P>0.05 | No | (3) |
| ANM | European | ReproGen (2015) | 70000 | CRP | European | - | - | 56 | IVW | 0.006 (-0.036 to 0.047) | P>0.05 | No | (3) |
| ANM | European | ReproGen (2015) | 69360 | BC | European | BCAC | 247173 | 53 | IVW | 1.09 (1.06 to 1.13) | 2.71*10-7 | Yes | (10) |
| ANM | European | ReproGen (2015) | 69626 | BC | European | BCAC | 86627 | 34 | IVW | 1.01 (0.98 to 1.05) | 0.49 | Yes | (11) |
| ANM | European | ReproGen (2015) | 69360 | BC | European | BCAC, DRIVE | 139274 | 41 | IVW | 1.054 (1.034 to 1.075) | 8.010*10-8 | Yes | (12) |
| ANM | European | ReproGen (2015) | 69360 | BC | European | BCAC, Discovery, DRIVE, iCOGS | 228951 | 42 | IVW | 1.05 (1.03 to 1.07) | P<0.001 | No | (13) |
| ANM | European | ReproGen (2021) | 201323 | BC | European | Large cohorts (Michailidou et al.) | 228951 | - | IVW | 0.96 (0.95 to 0.98) | P<0.05 | Yes | (7) |
| ANM | European | ReproGen (2021) | 201323 | BC | European | UKB | 181279 | - | One-sample MR | 0.94 (0.92 to 0.96) | P<0.05 | Yes | (7) |
| ANM | European | ReproGen (2021) | 201323 | BC | European | ReproGen (2021) | 201323 | 229 | IVW | 1.035 (1.027 to 1.029) | 3.7*10-17 | Yes | (2) |
| ANM | European | WHI | 106853 | BC | European | WHI | 17906 | - | One-sample MR | 1.82 (1.50 to 2.21) | P<0.05 | Yes | (4) |
| ANM | European | UKB | 95464 | BC | European | UKB | 91205 | - | One-sample MR | 0.98 (0.82 to 1.17) | P>0.05 | Yes | (4) |
| ANM | European | NHS | 2182 | BC | European | NHS | 2182 | - | One-sample MR | 1.59 (0.97 to 2.60) | P>0.05 | Yes | (4) |
| ANM | European | WHI+UKB | 109111 | BC | European | WHI+UKB | 109111 | - | One-sample MR | 1.33 (0.73 to 2.44) | P>0.05 | Yes | (4) |
| ANM | European | ALL | 111293 | BC | European | ALL | 111293 | - | One-sample MR | 1.40 (0.88 to 2.24) | P>0.05 | Yes | (4) |
| ANM | European | ReproGen (2015) | 69360 | ER+ BC | European | BCAC | 175475 | - | IVW | 1.11 (1.06 to 1.15) | 4.45*10-7 | Yes | (10) |
| ANM | European | ReproGen (2015) | 69626 | ER+ BC | European | BCAC | 59378 | - | IVW | 1.03 (0.98 to 1.08) | 0.21 | Yes | (11) |
| ANM | European | ReproGen (2015) | 69360 | ER+ BC | European | BCAC, Discovery, DRIVE, iCOGS | 66450 | 42 | IVW | 1.03 (1.00 to 1.06) | 0.051 | No | (13) |
| ANM | European | ReproGen (2021) | 201323 | ER+ BC | European | ReproGen (2021) | 201323 | 256 | IVW | 1.015 (1.002 to 1.029) | 0.021 | Yes | (2) |
| ANM | European | ReproGen (2015) | 69360 | ER- BC | European | BCAC | 127442 | - | IVW | 1.07 (1.02 to 1.12) | 0.005 | Yes | (10) |
| ANM | European | ReproGen (2015) | 69626 | ER- BC | European | BCAC | 13692 | - | IVW | 1.01 (0.93 to 1.08) | 0.9 | Yes | (11) |
| ANM | European | ReproGen (2015) | 69360 | ER- BC | European | BCAC, Discovery, DRIVE, iCOGS | 175475 | 42 | IVW | 1.05 (1.03 to 1.08) | P<5.85*10-5 | No | (13) |
| ANM | European | ReproGen (2021) | 201323 | ER- BC | European | ReproGen (2021) | 201323 | 227 | IVW | 1.041 (1.032 to 1.05) | 2.7*10-16 | Yes | (2) |
| ANM | European | ReproGen (2015) | 69360 | Luminal A like BC | European | BCAC | 136730 | - | IVW | 1.11 (1.06 to 1.16) | 2.14*10-6 | Yes | (10) |
| ANM | European | ReproGen (2015) | 69360 | Luminal B/HER2- BC | European | BCAC | 97827 | - | IVW | 1.11 (1.06 to 1.17) | 3.02*10-5 | Yes | (10) |
| ANM | European | ReproGen (2015) | 69360 | Luminal B BC | European | BCAC | 97904 | - | IVW | 1.08 (1.01 to 1.16) | 0.034 | Yes | (10) |
| ANM | European | ReproGen (2015) | 69360 | HER2 enriched BC | European | BCAC | 94361 | - | IVW | 1.13 (1.03 to 1.23) | 0.01 | Yes | (10) |
| ANM | European | ReproGen (2015) | 69360 | Triple- BC | European | BCAC | 100079 | - | IVW | 1.03 (0.97 to 1.09) | 0.407 | Yes | (10) |
| ANM | European | WHI | 13200 | EC | European | WHI | 13200 | - | One-sample MR | 2.37 (1.23 to 4.57) | P<0.05 | Yes | (4) |
| ANM | European | UKB | 95151 | EC | European | UKB | 95151 | - | One-sample MR | 1.37 (0.96 to 1.96) | P>0.05 | Yes | (4) |
| ANM | European | NHS | 3441 | EC | European | E2C2 | 3441 | - | One-sample MR | 0.89 (0.58 to 1.34) | P>0.05 | Yes | (4) |
| ANM | European | WHI+UKB | 108351 | EC | European | WHI+UKB | 108351 | - | One-sample MR | 1.68 (1.00 to 2.82) | P>0.05 | Yes | (4) |
| ANM | European | ALL | 111792 | EC | European | ALL | 111792 | - | One-sample MR | 1.35 (0.83 to 2.18) | P>0.05 | Yes | (4) |
| ANM | European | WHI | 17059 | OC | European | WHI | 17059 | - | One-sample MR | 1.19 (0.47 to 3.00) | P>0.05 | Yes | (4) |
| ANM | European | UKB | 95304 | OC | European | UKB | 95304 | - | One-sample MR | 1.39 (0.85 to 2.27) | P>0.05 | Yes | (4) |
| ANM | European | ALL | 112363 | OC | European | ALL | 112363 | - | One-sample MR | 1.35 (0.87 to 2.07) | P>0.05 | Yes | (4) |
| ANM | European | ReproGen (2015) | 69360 | OC | European | OCAC | 127442 | 42 | IVW | 1.04 (1.01 to 1.06) | P<0.05 | No | (13) |
| ANM | European | ReproGen (2021) | 201323 | OC | European | ReproGen (2021) | 201323 | 223 | IVW | 1.028 (1.013 to 1.043) | 2.9*10-4 | Yes | (2) |
| ANM | European | ReproGen (2015) | 69360 | Endometrioid OC | European | OCAC | 43751 | 42 | IVW | 1.10 (1.04 to 1.16) | P<0.05 | No | (13) |
| ANM | European | ReproGen (2015) | 69360 | Endometrioid OC | European | OCAC | 43751 | 35 | IVW | 1.09 (1.02 to 1.16) | 0.007 | Yes | (14) |
| ANM | European | ReproGen (2015) | 69360 | IEOC | European | OCAC | 63347 | 35 | IVW | 1.03 (1.00 to 1.06) | 0.07 | Yes | (14) |
| ANM | European | ReproGen (2015) | 69360 | High grade serous OC | European | OCAC | 53978 | 35 | IVW | 1.02 (0.99 to 1.05) | 0.31 | Yes | (14) |
| ANM | European | ReproGen (2015) | 69360 | Low grade serous OC | European | OCAC | 41953 | 35 | IVW | 1.01 (0.92 to 1.12) | 0.77 | Yes | (14) |
| ANM | European | ReproGen (2015) | 69360 | Mucinous OC | European | OCAC | 42358 | 35 | IVW | 1.00 (0.92 to 1.09) | 0.97 | Yes | (14) |
| ANM | European | ReproGen (2015) | 69360 | Clear cell OC | European | OCAC | 42307 | 35 | IVW | 1.05 (0.96 to 1.14) | 0.29 | Yes | (14) |
| ANM | European | ReproGen (2015) | 69360 | Low malignant potential OC | European | OCAC | 44044 | 35 | IVW | 1.04 (0.98 to 1.10) | 0.21 | Yes | (14) |
| ANM | European | ReproGen (2015) | 69360 | CRC | European | GECCO, CCFR, CORECT | 23685 | 51 | GRS-based analyses | 0.98 (0.94 to 1.01) | P>0.05 | Yes | (15) |
| ANM | European | ReproGen (2015) | 69360 | CRC | European | GECCO, CCFR, CORECT | 23685 | 51 | MR-Egger | 1.02 (0.94 to 1.10) | P>0.05 | Yes | (15) |
| ANM | European | ReproGen (2015) | 69360 | CRC | European | GECCO, CCFR, CORECT | 23685 | 51 | Weighted median estimator | 1.00 (0.95 to 1.05) | P>0.05 | Yes | (15) |
| ANM | European | WHI | 17981 | LC | European | WHI | 17981 | - | One-sample MR | 1.52 (0.96 to 2.41) | P>0.05 | Yes | (4) |
| ANM | European | UKB | 95390 | LC | European | UKB | 95390 | - | One-sample MR | 1.34 (0.94 to 1.89) | P>0.05 | Yes | (4) |
| ANM | European | EAGLE+ATBC+PLCO+CBP-II GWAS | 1285 | LC | European | EAGLE+ATBC+PLCO+CBP-II GWAS | 1285 | - | One-sample MR | 0.89 (0.47 to 1.68) | P>0.05 | Yes | (4) |
| ANM | European | NCI GWAS | 986 | LC | European | NCI GWAS | 986 | - | One-sample MR | 1.93 (0.83 to 4.48) | P>0.05 | Yes | (4) |
| ANM | European | High density SNP analysis | 741 | LC | European | High density SNP analysis | 741 | - | One-sample MR | 1.35 (0.51 to 3.55) | P>0.05 | Yes | (4) |
| ANM | European | WHI+UKB | 113371 | LC | European | WHI+UKB | 113371 | - | One-sample MR | 1.40 (1.06 to 1.85) | P<0.05 | Yes | (4) |
| ANM | European | All | 116383 | LC | European | All | 116383 | - | One-sample MR | 1.35 (1.06 to 1.71) | P<0.05 | Yes | (4) |
| ANM | European | ReproGen (2021) | 201323 | FVC | European | Large cohorts (Shrine et al.) | 79055 | - | IVW | -0.0067 (-0.0147 to 0.0013) | P>0.05 | Yes | (7) |
| ANM | European | ReproGen (2021) | 201323 | FVC | European | UKB | 181279 | - | One-sample MR | -0.0036 (-0.0093 to 0.0022) | P>0.05 | Yes | (7) |
| early ANM | European | UKB | 7206 | Spirometric restriction | European | UKB | 86274 | 39 | IVW | 0.29 (0.22 to 0.36) | 1.48*10-16 | Yes | (16) |
| early ANM | European | UKB | 7206 | Airflow obstruction | European | UKB | 86274 | 39 | IVW | 0.85 (0.82 to 0.89) | 5.88*10-14 | Yes | (16) |
| early ANM excluding height SNPs | European | UKB | 7206 | Spirometric restriction | European | UKB | 86274 | 30 | IVW | 0.28 (0.21 to 0.36) | 2.29*10-13 | Yes | (16) |
| early ANM excluding height SNPs | European | UKB | 7206 | Airflow obstruction | European | UKB | 86274 | 30 | IVW | 0.84 (0.81 to 0.88) | 1.44*10-13 | Yes | (16) |
| early excluding BMI SNPs | European | UKB | 7206 | Spirometric restriction | European | UKB | 86274 | 36 | IVW | 0.29 (0.22 to 0.36) | 1.93*10-16 | Yes | (16) |
| early excluding BMI SNPs | European | UKB | 7206 | Airflow obstruction | European | UKB | 86274 | 36 | IVW | 0.85 (0.82 to 0.89) | 7.60*10-14 | Yes | (16) |
| early ANM excluding menarche SNPs | European | UKB | 7206 | Spirometric restriction | European | UKB | 86274 | 36 | IVW | 0.29 (0.22 to 0.36) | 1.02*10-14 | Yes | (16) |
| early ANM excluding menarche SNPs | European | UKB | 7206 | Airflow obstruction | European | UKB | 86274 | 36 | IVW | 0.84 (0.81 to 0.88) | 2.85*10-14 | Yes | (16) |
| early ANM excluding all pleiotropic SNPs | European | UKB | 7206 | Spirometric restriction | European | UKB | 86274 | 28 | IVW | 0.29 (0.22 to 0.37) | 7.13*10-14 | Yes | (16) |
| early ANM excluding all pleiotropic SNPs | European | UKB | 7206 | Airflow obstruction | European | UKB | 86274 | 28 | IVW | 0.84 (0.80 to 0.88) | 1.19*10-13 | Yes | (16) |
| late ANM | European | UKB | 8468 | Spirometric restriction | European | UKB | 87536 | 40 | IVW | -0.18 (-0.26 to -0.10)] | 1.09*10-5 | Yes | (16) |
| late ANM | European | UKB | 8468 | Airflow obstruction | European | UKB | 87536 | 40 | IVW | 1.06 (1.01 to 1.11) | 0.018 | Yes | (16) |
| late ANM excluding height SNPs | European | UKB | 8468 | Spirometric restriction | European | UKB | 87536 | 31 | IVW | -0.22 (-0.33 to -0.11) | 6.90*10-5 | Yes | (16) |
| late ANM excluding height SNPs | European | UKB | 8468 | Airflow obstruction | European | UKB | 87536 | 31 | IVW | 1.10 (1.03 to 1.17) | 4.53*10-3 | Yes | (16) |
| late excluding BMI SNPs | European | UKB | 8468 | Spirometric restriction | European | UKB | 87536 | 37 | IVW | -0.17 (-0.26 to -0.09) | 3.13*10-5 | Yes | (16) |
| late excluding BMI SNPs | European | UKB | 8468 | Airflow obstruction | European | UKB | 87536 | 37 | IVW | 1.06 (1.01 to 1.11) | 0.027 | Yes | (16) |
| late ANM excluding menarche SNPs | European | UKB | 8468 | Spirometric restriction | European | UKB | 87536 | 37 | IVW | -0.23 (-0.33 to -0.13) | 7.32*10-6 | Yes | (16) |
| late ANM excluding menarche SNPs | European | UKB | 8468 | Airflow obstruction | European | UKB | 87536 | 37 | IVW | 1.11 (1.04 to 1.17) | 1.09*10-3 | Yes | (16) |
| late ANM excluding all pleiotropic SNPs | European | UKB | 8468 | Spirometric restriction | European | UKB | 87536 | 28 | IVW | -0.22 (-0.33 to -0.11) | 1.33*10-4 | Yes | (16) |
| late ANM excluding all pleiotropic SNPs | European | UKB | 8468 | Airflow obstruction | European | UKB | 87536 | 28 | IVW | 1.09 (1.02 to 1.17) | 0.015 | Yes | (16) |
| ANM | European | WHI | 17981 | Fracture | European | WHI | 17981 | - | One-sample MR | 0.72 (0.58 to 0.89) | P<0.05 | Yes | (4) |
| ANM | European | UKB | 95258 | Fracture | European | UKB | 95258 | - | One-sample MR | 0.94 (0.60 to 1.48) | P>0.05 | Yes | (4) |
| ANM | European | All | 113239 | Fracture | European | All | 113239 | - | One-sample MR | 0.76 (0.62 to 0.94) | P<0.05 | Yes | (4) |
| ANM | European | ReproGen (2015) | 69360 | Fracture risk | Most European | GEFOS | 297285 | 54 | IVW | 1.10 (1.00 to 1.21) | 0.05 | Yes | (17) |
| ANM | European | ReproGen (2021) | 201323 | Fracture risk | European | ReproGen (2021) | 201323 | 212 | IVW | 0.983 (0.974 to 0.992) | 4.7*10-4 | Yes | (2) |
| ANM | European | ReproGen (2021) | 201323 | BMD (45-60 years) | European | ReproGen (2021) | 201323 | 243 | IVW | 0.033 (0.021 to 0.045) | 6.9*10-8 | Yes | (2) |
| ANM | European | ReproGen (2021) | 201323 | FNBMD | European | FNBMD 2012 | ～22000 | - | IVW | -0.04 (-0.16 to 0.08) | P>0.05 | Yes | (7) |
| ANM | European | ReproGen (2021) | 201323 | LSBMD | European | LSBMD Estrada 2012 | ～22000 | - | IVW | -0.05 (-0.07 to -0.03) | P<0.05 | Yes | (7) |
| ANM | European | ReproGen (2021) | 201323 | HeelBMD | European | UKB | 181279 | - | One-sample MR | -0.02 (-0.03 to -0.02) | P<0.05 | Yes | (7) |
| ANM | European | WHI | 17960 | Osteoporosis | European | WHI | 17960 | - | One-sample MR | 0.95 (0.79 to 1.14) | P>0.05 | Yes | (4) |
| ANM | European | UKB | 91547 | Osteoporosis | European | UKB | 91547 | - | One-sample MR | 0.74 (0.64 to 0.86) | P<0.05 | Yes | (4) |
| ANM | European | Large cohorts (Kaiser et al.) | 27573 | Osteoporosis | European | Large cohorts (Kaiser et al.) | 27573 | - | One-sample MR | 0.76 (0.63 to 0.92) | P<0.05 | Yes | (4) |
| ANM | European | WHI+UKB | 109507 | Osteoporosis | European | WHI+UKB | 109507 | - | One-sample MR | 0.84 (0.66 to 1.06) | P>0.05 | Yes | (4) |
| ANM | European | All | 137080 | Osteoporosis | European | All | 137080 | - | One-sample MR | 0.81 (0.69 to 0.94) | P<0.05 | Yes | (4) |
| ANM | European | ReproGen (2015) | 69360 | OA | European | UKB | 327918 | 51 | IVW | 1.00 (0.97 to 1.03) | 0.905 | Yes | (18) |
| ANM | European | ReproGen (2015) | 69360 | Hip OA | European | UKB | 327918 | 51 | IVW | 0.98 (0.92 to 1.03) | 0.443 | Yes | (18) |
| ANM | European | ReproGen (2015) | 69360 | Knee OA | European | UKB | 327918 | 51 | IVW | 1.00 (0.96 to 1.04) | 0.946 | Yes | (18) |
| ANM | European | ReproGen (2015) | 69360 | Hip and/or knee OA | European | UKB | 327918 | 51 | IVW | 1.00 (0.97 to 1.04) | 0.795 | Yes | (18) |
| ANM | European | ReproGen (2015) | 69360 | RA | European | Large cohorts (Okada et al.) | 58284 | 54 | IVW | 1.05 (0.98 to 1.11) | 0.15 | Yes | (19) |
| ANM removing palindromic SNPs | European | ReproGen (2015) | 69360 | RA | European | Large cohorts (Okada et al.) | 58284 | 47 | IVW | 1.05 (0.98 to 1.13) | 0.13 | Yes | (19) |
| ANM removing confounding SNPs | European | ReproGen (2015) | 69360 | RA | European | Large cohorts (Okada et al.) | 58284 | 42 | IVW | 1.04 (0.95 to 1.14) | 0.4 | Yes | (19) |
| ANM adjusting for BMI | European | ReproGen (2015) | 69360 | RA | European | Large cohorts (Okada et al.) | 58284 | 51 | MVMR | 1.06 (0.99 to 1.27) | 0.08 | Yes | (19) |
| ANM adjusting for year of education | European | ReproGen (2015) | 69360 | RA | European | Large cohorts (Okada et al.) | 58284 | 54 | MVMR | 1.04 (0.98 to 1.11) | 0.18 | Yes | (19) |
| ANM | European | WHI | 14740 | AD | European | WHI | 14740 | - | One-sample MR | 1.06 (0.73 to 1.54) | P>0.05 | Yes | (4) |
| ANM | European | UKB | 95462 | AD | European | UKB | 95462 | - | One-sample MR | 0.97 (0.65 to 1.45) | P>0.05 | Yes | (4) |
| ANM | European | ALL | 110202 | AD | European | ALL | 110202 | - | One-sample MR | 1.02 (0.77 to 1.34) | P>0.05 | Yes | (4) |
| ANM without outliers | European | ReproGen (2015) | 69360 | AD | European | IGAP | 54162 | 23 | IVW | 0.975 (0.935 to 1.017) | 0.241 | Yes | (9) |
| ANM with outliers | European | ReproGen (2015) | 69360 | AD | European | IGAP | 54162 | 38 | IVW | 0.991 (0.957 to 1.026) | 0.611 | Yes | (9) |
| ANM | European | ReproGen (2015) | 69360 | Cognitive performance | European | IGAP | 257841 | 29 | IVW | -0.002 (-0.008 to 0.005) | 0.585 | Yes | (9) |
| ANM | European | PEG, PASIDA | 2260 | PD | European | PEG, PASIDA | 2260 | 8 | IVW | 0.85 (0.73 to 0.98) | 0.03 | Yes | (20) |
| ANM | European | IPDGC excluding UKB | 19773 | PD | European | IPDGC excluding UKB | 19773 | 8 | IVW | 0.94 (0.90 to 0.99) | 0.01 | Yes | (20) |
| ANM | European | ReproGen (2021) | 201323 | aSAH | European | Large cohorts (Bakker et al.) | 77074 | 112 | IVW | 1.01 (0.96 to 1.06) | 0.807 | Yes | (21) |
| ANM | European | Rotterdam, 23andMe Study | 94926 | PCOS | European | Rotterdam, 23andMe Study | 94926 | - | One sample MR | 1.60 (1.35-1.91) | 1.50*10-8 | NR | (22) |
| ANM | European | European | Rotterdam, UK (London/Oxford), EGCUT, deCODE, Chicago, Bostan, 23andMe study | PCOS | European | Rotterdam, UK (London/Oxford), EGCUT, deCODE, Chicago, Bostan, 23andMe study | 113238 | - | One sample MR | 0.1 (Beta) | 1.31*10-5 | Yes | (23) |
| ANM | European | WHI | 38968 | Acceleration epigenetic aging | European | WHI | 1940 | 1 (rs11668344) | Ward ratio | 0.506 | 0.031 | NR | (24) |
| ANM | European | WHI | 38968 | Acceleration epigenetic aging | European | WHI | 1940 | 1 (rs16991615) | Ward ratio | 0.151 | 0.763 | NR | (24) |
| ANM | European | ReproGen (2021) | 201323 | ALP | European | Large cohorts (Chambers et al.) | 61089 | - | IVW | -0.0005 (-0.0034 to 0.0024) | P>0.05 | Yes | (7) |
| ANM | European | ReproGen (2021) | 201323 | ALP | European | UKB | 181279 | - | One-sample MR | -0.0019 (-0.0033 to -0.0005) | P>0.05 | Yes | (7) |
| ANM | European | ReproGen (2021) | 201323 | ALT | European | Large cohorts (Chambers et al.) | 61089 | - | IVW | 0.0004 (-0.0037 to 0.0046) | P>0.05 | Yes | (7) |
| ANM | European | ReproGen (2021) | 201323 | ALT | European | UKB | 181279 | - | One-sample MR | -0.0010 (-0.0022 to 0.0003) | P>0.05 | Yes | (7) |
| ANM | European | ReproGen (2021) | 201323 | Creatinine | European | Large cohorts (Pattaro et al.) | 133413 | - | IVW | 0.000 (-0.002 to 0.002) | P>0.05 | Yes | (7) |
| ANM | European | ReproGen (2021) | 201323 | Creatinine | European | UKB | 181279 | - | One-sample MR | -0.001 ('-0.002 to -0.001) | P>0.05 | Yes | (7) |
| ANM | European | ReproGen (2021) | 201323 | Urea | European | Large cohorts (Pattaro et al.) | 133413 | - | IVW | -0.009 (-0.021 to 0.004) | P>0.05 | Yes | (7) |
| ANM | European | ReproGen (2021) | 201323 | Urea | European | UKB | 181279 | - | One-sample MR | -0.005 (-0.005 to -0.004) | P>0.05 | Yes | (7) |
| ANM | European | ReproGen (2021) | 201323 | Coeliac disease | European | Large cohorts (Dubois et al.) | 24269 | - | IVW | 1.01 (0.95 to 1.07) | P>0.05 | Yes | (7) |
| ANM | European | ReproGen (2021) | 201323 | Coeliac disease | European | UKB | 181279 | - | One-sample MR | 1.17 (1.10 to 1.23) | P<0.05 | Yes | (7) |
| ANM | European | UKB | 143791 | AAM | European | UKB | 243898 | 84 | IVW | 0.039 (0.021 to 0.062) | 0.08068211 | Yes | (25) |
| ANM | European | UKB | 143791 | AFS | European | UKB | 219486 | 84 | IVW | 0.025 (0.013 to 0.063) | 0.05140582 | Yes | (25) |
| ANM | European | UKB | 143791 | AFB | European | UKB | 203606 | 84 | IVW | 0.0248 (0.015 to 0.100) | 0.0544792 | Yes | (25) |
| ANM | European | UKB | 143791 | ALB | European | UKB | 203356 | 84 | IVW | 0.025 (-0.002 to 0.052) | 0.07474091 | Yes | (25) |
| ANM | European | UKB | 143791 | NLB | European | UKB | 250746 | 84 | IVW | -0.018 (-0.043 to 0.007) | 0.151489 | Yes | (25) |
| ANM | European | UKB | 143791 | LNS | European | UKB | 208274 | 84 | IVW | 0.016 (-0.012 to 0.044) | 0.2609884 | Yes | (25) |
| ANM | European | UKB | 143791 | EPS | European | UKB | 250746 | 84 | IVW | 0.996 (0.986 to 1.007) | 0.4761175 | Yes | (25) |

Note: SNP, single nucleotide polymorphisms; MR, mendelian randomization; OR, odds ratio; ANM, age at menopause; AF, atrial fibrillation; HUNT, the Nord-Trondelag Health Study; MGI, the Michigan Genomics Initiative; UKB, UK Biobank; IVW, inverse-variance weighted; CAD, coronary artery disease; HF, heart failure; HERMES, Heart Failure Molecular Epidemiology for Therapeutic Targets Consortium; IS, ischemic stroke; MEGASTROKE, a large-scale international collaboration launched by the International Stroke Genetics Consortium; CHD, coronary heart disease; DDR, DNA damage response; GLGC, Global Lipid Genetics Consortium; HDL, high-density lipoprotein; HbA1c, glycosylated hemoglobin; MAGIC, the Meta-Analyses of Glucose and Insulin-related traits Consortium; CRP, C-reactive protein; ApoA1, apolipoprotein A1; ApoB, apolipoprotein B; SBP, systolic blood pressure; DBP, diastolic blood pressure; T2DM, type 2 diabetes mellitus; ER, estrogen receptor; BC, breast cancer; BCAC, Breast Cancer Association Consortium; DRIVE, Biology and Risk of Inherited Variants in Breast Cancer Consortium; WHI, Women Health Initiative; NHS, Nurses Health Study; EC, endometrial cancer; E2C2, Epidemiology of Endometrial Cancer Consortium; High density SNP analysis, High Density SNP Association Analysis of Lung Cancer; OC, ovarian cancer; LC, lung cancer; PLCO+CBP-II, Prostate, Colon, Ovary Screening trial + Cancer Prevention Study II; NCI GWAS, National Cancer Institute Genome Consortium Association Study of Lung Cancer in Never Smokers; CHS, Cardiovascular Health Study; ARIC, Atherosclerosis Risk in the Community Study; FHS, Framingham Heart Study; MESA, Multi-Ethnic Study of Atherosclerosis; WTCCC2, Wellcome Trust Case Consortium 2; iCOGS, Collaborative Oncological Gene-environment Study; OCAC, the Ovarian Cancer Association Consortium; IEOC, invasive epithelial ovarian cancer; PD, Parkinson’s disease; PEG, the Parkinson's Environment and Gene study; PASIDA, the Parkinson's disease in Denmark study; AD, Alzheimer’s disease; IPDGC, the International Parkinson's Disease Genomics Consortium; IGAP, the International genomics of Alzheimer's Project; GIANT, the Genetic Investigation of Anthropometric Traits consortium; BMI, body mass index; ALP, alkaline phosphatase; ALT, alanine transaminase; LDL, low-density lipoprotein; HbA1c, glycosylated hemoglobin; FVC, forced vital capacity; FNBMD, femoral neck bone-mineral density; LSBMD, lumber spine bone-mineral density; BMD, bone-mineral density; aSAH, aneurysmal subarachnoid hemorrhage; PCOS, polycystic ovarian syndrome; CRC, colorectal cancer; GECCO, the Genetics and Epidemiology of Colorectal Cancer Consortium; CCFR, the Colon Cancer Family Registry; CORECT, the Colorectal Cancer Transdisciplinary Consortium; AAM, age at menarche; AFS, age at first sexual intercourse; AFB, age at first live birth; ALB, age at last live birth; NLB, number of live births; LNS, lifetime number of sexual partners; EPS, ever parous status; GEFOS, the Genetic Factor for Osteoporosis consortium; DIAGRAM, the Diabetes Genetics Replication and Meta-analysis consortium; FI, fasting insulin; MAGIC, the Glucose and Insulin-Related Traits Consortium; FPG, fasting plasma glucose; HOMA-B, homeostasis model of B-cell function; HOMA-IR, homeostasis model of insulin resistance; RA, rheumatoid arthritis; OA, osteoarthritis.

**References**

1. Ardissino, M., Slob, E.A.W., Carter, P., Rogne, T., Girling, J., Burgess, S., et al. Sex-Specific Reproductive Factors Augment Cardiovascular Disease Risk in Women: A Mendelian Randomization Study. *J Am Heart Assoc* (2023) 12(5):e027933. doi: 10.1161/jaha.122.027933.

2. Ruth, K.S., Day, F.R., Hussain, J., Martínez-Marchal, A., Aiken, C.E., Azad, A., et al. Genetic insights into biological mechanisms governing human ovarian ageing. *Nature* (2021) 596(7872):393-7. doi: 10.1038/s41586-021-03779-7.

3. Dam, V., Onland-Moret, N.C., Burgess, S., Chirlaque, M.D., Peters, S.A.E., Schuit, E., et al. Genetically Determined Reproductive Aging and Coronary Heart Disease: A Bidirectional 2-sample Mendelian Randomization. *J Clin Endocrinol Metab* (2022) 107(7):e2952-e61. doi: 10.1210/clinem/dgac171.

4. Lankester, J., Li, J., Salfati, E.L.I., Stefanick, M.L., Chan, K.H.K., Liu, S., et al. Genetic evidence for causal relationships between age at natural menopause and the risk of ageing-associated adverse health outcomes. *Int J Epidemiol* (2022). doi: 10.1093/ije/dyac215.

5. Wang, Z., Lu, J., Weng, W., Zhang, L., Zhang, J. Women's reproductive traits and ischemic stroke: a two-sample Mendelian randomization study. *Ann Clin Transl Neurol* (2023) 10(1):70-83. doi: 10.1002/acn3.51702.

6. Roa-Díaz, Z.M., Asllanaj, E., Amin, H.A., Rojas, L.Z., Nano, J., Ikram, M.A., et al. Age at Natural Menopause and Blood Pressure Traits: Mendelian Randomization Study. *J Clin Med* (2021) 10(19). doi: 10.3390/jcm10194299.

7. Magnus, M.C., Borges, M.C., Fraser, A., Lawlor, D.A. Identifying potential causal effects of age at menopause: a Mendelian randomization phenome-wide association study. *Eur J Epidemiol* (2022) 37(9):971-82. doi: 10.1007/s10654-022-00903-3.

8. Xing, W., Lv, Q., Li, Y., Wang, C., Mao, Z., Li, Y., et al. Genetic prediction of age at menarche, age at natural menopause and type 2 diabetes: A Mendelian randomization study. *Nutr Metab Cardiovasc Dis* (2023) 33(4):873-82. doi: 10.1016/j.numecd.2023.01.011.

9. Li, M., Lin, J., Liang, S., Chen, Z., Bai, Y., Long, X., et al. The role of age at menarche and age at menopause in Alzheimer's disease: evidence from a bidirectional mendelian randomization study. *Aging (Albany NY)* (2021) 13(15):19722-49. doi: 10.18632/aging.203384.

10. Chen, F., Wen, W., Long, J., Shu, X., Yang, Y., Shu, X.O., et al. Mendelian randomization analyses of 23 known and suspected risk factors and biomarkers for breast cancer overall and by molecular subtypes. *Int J Cancer* (2022) 151(3):372-80. doi: 10.1002/ijc.34026.

11. Escala-Garcia, M., Morra, A., Canisius, S., Chang-Claude, J., Kar, S., Zheng, W., et al. Breast cancer risk factors and their effects on survival: a Mendelian randomisation study. *BMC Med* (2020) 18(1):327. doi: 10.1186/s12916-020-01797-2.

12. Jia, L., Lv, W., Liang, L., Ma, Y., Ma, X., Zhang, S., et al. The Causal Effect of Reproductive Factors on Breast Cancer: A Two-Sample Mendelian Randomization Study. *J Clin Med* (2023) 12(1). doi: 10.3390/jcm12010347.

13. Si, S., Li, J., Tewara, M.A., Li, H., Liu, X., Li, Y., et al. Identifying causality, genetic correlation, priority and pathways of large-scale complex exposures of breast and ovarian cancers. *Br J Cancer* (2021) 125(11):1570-81. doi: 10.1038/s41416-021-01576-7.

14. Yarmolinsky, J., Relton, C.L., Lophatananon, A., Muir, K., Menon, U., Gentry-Maharaj, A., et al. Appraising the role of previously reported risk factors in epithelial ovarian cancer risk: A Mendelian randomization analysis. *PLoS Med* (2019) 16(8):e1002893. doi: 10.1371/journal.pmed.1002893.

15. Neumeyer, S., Banbury, B.L., Arndt, V., Berndt, S.I., Bezieau, S., Bien, S.A., et al. Mendelian randomisation study of age at menarche and age at menopause and the risk of colorectal cancer. *Br J Cancer* (2018) 118(12):1639-47. doi: 10.1038/s41416-018-0108-8.

16. van der Plaat, D.A., Pereira, M., Pesce, G., Potts, J.F., Amaral, A.F.S., Dharmage, S.C., et al. Age at menopause and lung function: a Mendelian randomisation study. *Eur Respir J* (2019) 54(4). doi: 10.1183/13993003.02421-2018.

17. Trajanoska, K., Morris, J.A., Oei, L., Zheng, H.F., Evans, D.M., Kiel, D.P., et al. Assessment of the genetic and clinical determinants of fracture risk: genome wide association and mendelian randomisation study. *Bmj* (2018) 362(k3225. doi: 10.1136/bmj.k3225.

18. Wang, B., Wu, J., Li, H., Jin, X., Sui, C., Yu, Z. Using genetic instruments to estimate the causal effect of hormonal reproductive factors on osteoarthritis. *Front Public Health* (2022) 10(941067. doi: 10.3389/fpubh.2022.941067.

19. Zhu, J., Niu, Z., Alfredsson, L., Klareskog, L., Padyukov, L., Jiang, X. Age at menarche, age at natural menopause, and risk of rheumatoid arthritis - a Mendelian randomization study. *Arthritis Res Ther* (2021) 23(1):108. doi: 10.1186/s13075-021-02495-x.

20. Kusters, C.D.J., Paul, K.C., Duarte Folle, A., Keener, A.M., Bronstein, J.M., Bertram, L., et al. Increased Menopausal Age Reduces the Risk of Parkinson's Disease: A Mendelian Randomization Approach. *Mov Disord* (2021) 36(10):2264-72. doi: 10.1002/mds.28760.

21. Molenberg, R., Thio, C.H.L., Aalbers, M.W., Uyttenboogaart, M., Larsson, S.C., Bakker, M.K., et al. Sex Hormones and Risk of Aneurysmal Subarachnoid Hemorrhage: A Mendelian Randomization Study. *Stroke* (2022) 53(9):2870-5. doi: 10.1161/strokeaha.121.038035.

22. Day, F.R., Hinds, D.A., Tung, J.Y., Stolk, L., Styrkarsdottir, U., Saxena, R., et al. Causal mechanisms and balancing selection inferred from genetic associations with polycystic ovary syndrome. *Nat Commun* (2015) 6(8464. doi: 10.1038/ncomms9464.

23. Day, F., Karaderi, T., Jones, M.R., Meun, C., He, C., Drong, A., et al. Large-scale genome-wide meta-analysis of polycystic ovary syndrome suggests shared genetic architecture for different diagnosis criteria. *PLoS Genet* (2018) 14(12):e1007813. doi: 10.1371/journal.pgen.1007813.

24. Levine, M.E., Lu, A.T., Chen, B.H., Hernandez, D.G., Singleton, A.B., Ferrucci, L., et al. Menopause accelerates biological aging. *Proc Natl Acad Sci U S A* (2016) 113(33):9327-32. doi: 10.1073/pnas.1604558113.

25. Prince, C., Sharp, G.C., Howe, L.D., Fraser, A., Richmond, R.C. The relationships between women's reproductive factors: a Mendelian randomisation analysis. *BMC Med* (2022) 20(1):103. doi: 10.1186/s12916-022-02293-5.
